# Medical oxygen and respiratory support requirements for patients hospitalised with COVID-19 in 23 low-income and middle-income countries: a prospective, observational cohort study

**DOI:** 10.1016/S2214-109X(25)00480-2

**Published:** 2026-07-01

**Authors:** Pryanka Relan, Jamie Rylance, Yaseen M Arabi, Pauline Convocar, Matthieu Rolland, Janet V Diaz, O2CoV2 investigators, Islam Gamal Albayadi, Ahmad Al-Touny, Aiman Al-Touny, Shimaa Ahmed Hamed Al-Touny, Carlos Arturo Alvarez-Moreno, Gasim Amrahli, Zeina Aoun Bacha, Masooma Aqeel, Yaseen Arabi, Diptesh Aryal, Hope Atuhaire, Celine Baaklini, Barnabas Bakamutumaho, Debashis Basu, Neale Batra, Abigail Beane, Katrina Bentulan, Benilde Bepouka, Anil Bilimale, Nguyen Thien Binh, Kieran Bligh, Ina Bolocan, Guillermo Caceres-Cadenas, Luis Alberto Camputaro, Itziar Carrasco-Garcia, Pauline Convocar, Monica Cruz, Matthew Cummings, Shaimaa Dahshan, Ichinnorov Dashtseren, Cinzia De Brito Procopio, Mohammed Derow, Jaidev Devadas, Christopher Devasahayam, Janet Diaz, Sara Domínguez-Rodríguez, Alejandro Jose Duarte Cuellar, Ryenchindorj Erkhembayar, Mario Fernando Escobar, Adeniyi Francis Fagbamigbe, Aniruddha Ghose, Delmy Virginia Granados Castro, Bridget Griffith, Christophe Guitton, Nicole Haber, Bassem Habr, Priscilla Haguma, Rashan Haniffa, Madiha Hashmi, Than Manh Hung, Shevin Jacob, Leticia Kawano, Shirish KC, Muhammad Haroon Khan, Zohair Ahmed Khan, Basheer Khassawneh, Khalid Kheirallah, Francis Kiweewa, Mark Kizito, Chamira Kodippily, Richard Kojan, Ashok Kumar, Arthur Kwizera, Juan Carlos Llontop Otero, Nombulelo Magula, Ata Mahmoodpoor, Jean Robert Makulo, Undram Mandakh, John C Marshall, John Mathabathe, Rozelyn D Reyes-Mauro, Rabiul Alam Md Erfan Uddin, Carlos Medina, Maria Mendes, Martin Meremikwu, Faith Joan Gaerlan, Deebya Raj Mishra, Srinivas Murthy, Kenneth Doya Nonesa, Sharon Nyesiga, Mulinda Nyirenda, Chimedsuren Ochir, Alina Ogizbayeva, Adejumobi Oluwabukola, Bernard Omech, Darius Owachi, Parash Pandey, Luigi Pisani, Sanjeev Rai, Gayle Alyannah A. Ramos-Lee, Sumayyah Rashan, Bassma Raslan, Pryanka Relan, Ingrid Lara, Carlos Alfonso Reyes Silva, Moussa Riachi, Elisabeth Riviello, Jose Antonio Rojas Gambasica, Matthieu Rolland, Jamie Rylance, Sairah Sadaf, Maximiliano Ivan Sánchez, Amadou Seck, Kiran Shetty, Hassan Soleimanpour, Elizabeth Stanway, Yedilbayeva Tanzira, Eman Teema, Dennis Teo, Louise Thwaites, Le Mau Toan, Khanyisile Tshabalala, Cesar Ugarte-Gil, Benedict Edward Valdez, Laura Alejandra Velez Ruiz Gaitan, Valdilea G. Veloso, Katerine Milagros Villaizan Paliza, Julie Viry, Wangari Waweru, Prashanth Y M, Marija Zdravkovic, Milena Zivanovic

**Affiliations:** a World Health Organization, Geneva, Switzerland; b King Saud bin Abdulaziz University for Health Sciences, Riyadh, Saudi Arabia; c Southern Philippines Medical Center, Davao City, Philippines

## Abstract

**Background:**

The COVID-19 pandemic highlighted a global shortage of, and inequity of access to, medical oxygen. Understanding patient outcomes and the capacities of health facilities to provide respiratory support including oxygen is key to matching need and demand. We report results from a global study including 23 low-income and middle-income countries.

**Methods:**

For this prospective, observational cohort study, consecutive patients aged 12 years or older with suspected or confirmed COVID-19 and evidence of respiratory distress were prospectively recruited within 24 h of hospital admission. Hospitals from 23 low-income and middle-income countries were included, representing all WHO regions. Baseline demographic and clinical data were collected, and daily follow-ups were recorded for in-hospital outcomes and respiratory support types. At the facility level, we assessed sources of oxygen and electricity, infrastructural and staffing capacity for critical care provision, and the capabilities of the facility for advanced respiratory support. The primary outcome was 30-day in-hospital mortality. This study was registered on ClinicalTrials.gov (NCT04918875).

**Findings:**

Between Jan 24 and Nov 22, 2022, 56 sites took part. Of 53 726 patients screened, 3070 were enrolled. 1814 (61·6%) of 2947 patients had two or more underlying medical conditions and initially received oxygen through nasal cannula or non-rebreather face masks with reservoir. Invasive mechanical ventilation was most frequently used in patients recruited in the Americas (75 [26·4%] of 284 patients) and in the Eastern Mediterranean (90 [18·0%] of 499 patients). The overall mortality was 649 (23·4%) of 2779 patients, varying by region from 53 (10·5%) of 506 patients in South-East Asia to 286 (37·6%) of 760 patients in Africa. Mortality was associated with the maximum level of respiratory support received: from 17 (8·6%) of 198 patients who received no oxygen, 99 (38·4%) of 258 patients for non-rebreather reservoir bags, and 205 (62·9%) of 326 for invasive ventilation.

**Interpretation:**

The availability and use of oxygen support options in low-income and middle-income countries are highly variable but appear significantly less in the African region. Mortality might be associated with a lack of access to oxygen, which varied across WHO regions but was highest in Africa. Despite many lessons learned from the COVID-19 pandemic, inequity in access to medical oxygen remains a challenge that WHO and partners must address in the post-pandemic era to avoid preventable deaths.

**Funding:**

UNITAID.

## Introduction

Despite its recognition as an essential medicine by WHO,^1^ an estimated 60% of the world's population do not have reliable access to affordable, high-quality medical oxygen.^2^ Hypoxaemia is a strong predictor of mortality in acute illness,^3^ and facility-level oxygen-improvement interventions have been shown to reduce mortality among acutely ill children.^4^ However, the medical oxygen ecosystem is complex, geographically varied, and multi-sectoral. The provision of effective oxygen therapy requires a secure supply chain for production and transport, prompt recognition of hypoxaemia, and appropriate oxygen delivery and patient monitoring.

Inequitable access means that, in low-income and middle-income countries (LMICs), fewer than one in three patients who require medical oxygen are adequately treated with it.^2^ In addition to the 374 million people who require medical oxygen each year, an estimated 52 million people with COVID-19 needed oxygen treatment in 2021, consuming 1·9 billion normal cubic metres (Nm^3^). This demand was in addition to the usual 1·2 billion Nm^3^ required annually for emergency and acute medical care, critical illness, and safe perioperative practice. The 2023 World Health Assembly resolution on Increasing Access to Medical Oxygen marked international recognition of the scale of the challenge.^5^ In parallel to increasing production capacity to meet baseline and surge needs, the resolution requested WHO to collect and analyse data on access to medical oxygen in health systems, share best practices, highlight priority medical devices, and establish a research agenda as needed.

**Research in context**

**Evidence before this study**

On March 1, 2025, we searched PubMed for articles published since Jan 1, 2020, with keywords “COVID-19” and “oxygen” as major terms, combined with “availability” or “limitation”. There were no language exclusions. The high demand for medical oxygen during the COVID-19 pandemic highlighted the disparities in the availability of oxygen as a medical gas and the availability of respiratory support devices, with major effects on preventable mortality. Despite global attention, data on oxygen availability and use of respiratory support devices in low-income and middle-income countries are generally limited to small, retrospective, single-centre, or local studies. A study of more than 66 000 patients treated with advanced respiratory support devices found that invasive mechanical ventilation was used more frequently and high-flow nasal oxygen less frequently in patients treated in low-income and middle-income countries compared with high-income countries. However, the study did not include patients without access to monitoring devices or oxygen therapy.

**Added value of this study**

This study provides a global overview of the availability and use of oxygen support options, focusing on the variable availability of devices, staff, and infrastructure across countries and their association with patient outcomes.

**Implications of all the available evidence**

Inequity in access to oxygen remains a global challenge. This study provides a reference point for the extent and implications of this inequity and should serve as an impetus for future research focused on understanding the factors and interventions that can be feasibly and sustainably implemented in low-income and middle-income countries. Lessons from the COVID-19 pandemic provide opportunities for WHO and partners to develop strategies to avoid preventable deaths.

Data are particularly crucial to the understanding of how and when medical oxygen is administered.^6^ Treatment options are expanding, with increasing availability of advanced non-invasive respiratory support such as high-flow nasal oxygen (HFNO), continuous positive airway pressure (CPAP), and non-invasive mechanical ventilation (NIV). However, use of these treatment options is constrained by availability, user knowledge, and a scarcity of robust data on their optimal use.^7^

The multinational observational study of patients with COVID-19 we report here aimed to describe the requirement for medical oxygen at the patient level and facility level in LMICs, the administration and delivery methods, and their association with patient outcomes. The results are intended to inform a globally relevant clinical trial of respiratory support strategies in patients with acute hypoxaemic respiratory failure.

## Methods

### Study design and participants

**Figure 1:**
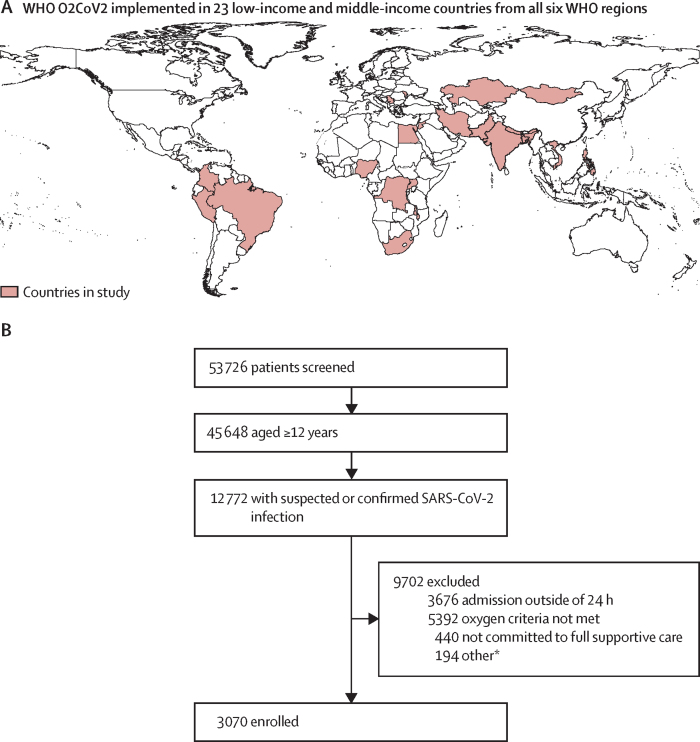
Participating countries (A) and study flowchart (B) Number of facilities in each country are given in parentheses: Democratic Republic of the Congo (n=1); Malawi (n=4); Nigeria (n=4); Uganda (n=4); South Africa (n=4); Brazil (n=1); Colombia (n=1); Peru (n=2); El Salvador (n=2); Egypt (n=1); Iran (n=1); Jordan (n=2); Lebanon (n=2); Pakistan (n=6); Kazakhstan (n=3); Moldova (n=2); Serbia (n=1); Bangladesh (n=3); India (n=4); Nepal (n=3); Mongolia (n=2); the Philippines (n=3); and Viet Nam (n=3). *Other reasons for exclusion include interrupted screening and enrolment due to patient condition, or lack of staff or functional equipment.

This prospective, observational cohort study was conducted at 56 sites across 23 LMICs (figure 1A). Eligible study sites were hospitals and temporary COVID-19 treatment centres that cared for patients with severe and critical COVID-19 and were able to screen patients continuously (day and night). Following an open request for expressions of interest to identify potential sites (n=211 responded to the initial survey), stratification was done firstly at the WHO regional level, and then in the Americas and Africa in subregional strata. Within this frame, sites were selected for inclusion by an independent selection committee according to whether they had dedicated research staff (to avoid redirection of clinical resources to research), and to whether a site could include an urban and rural facility (to improve representation and support research capacity building). Within each stratum, potential sites that met these criteria were selected randomly, with a maximum of two per country. Following a standardised process, the study steering committee selected facilities to ensure regional and subregional representation of LMICs.^7^ WHO headquarters COVID-19 Ethics Review Board approved the global protocol (CERC0040), which did not require written patient consent; however, written or oral patient consent was obtained as required by the relevant local authority. Local or national ethics approvals were obtained for each site. This study adhered to Good Clinical Practice and to the STROBE reporting guidelines.

Patients aged 12 years and older were recruited from emergency departments or hospital wards within 24 h of admission. Potential participants had either suspected COVID-19 (as assessed by the treating clinical provider), or a SARS-CoV-2 infection confirmed with RT-PCR testing of nasopharyngeal or oropharyngeal sample or rapid antigen testing according to local implementation. Patients were eligible for inclusion if they were receiving medical oxygen or had clinical evidence of the need for supplemental respiratory support (ie, one or more of respiratory rate ≥30 per min; oxygen saturation [SpO_2_] ≤90%; or SpO_2_ <94% with any emergency sign [eg, obstructed or absent breathing, severe respiratory distress, central cyanosis, shock, coma, or convulsions]). Patients were included only if they were receiving full supportive care (ie, not on palliative or end-of-life measures). Follow-up was continued daily until day 30, hospital discharge, or death, whichever occurred first.

### Procedures

Pulse oximeters and data collection tablet computers were provided to study sites by WHO. Data collectors received structured training on the data tools and definitions. Demographic data were collected at baseline. Sex data were collected from the patient's hospital file or self-report (recorded as male, female, or non-specified). Race data were not collected. Daily clinical follow-up was done on days 1–7 from admission and admission status and oxygen administration were recorded daily up to day 30. Data on comorbidity were extracted from patient records (handheld or institutional), or self-reported according to availability.

### Statistical analysis

The primary outcome was 30-day in-hospital mortality. The study protocol and statistical analysis plan have been published,^8^ and the full dataset and code are available online.^9^ Summary descriptive statistics were given for continuous variables as medians (IQR) or means (SD) as appropriate, and categorical variables as frequencies with percentages. For associations with 30-day hospital mortality, a Cox mixed effects model incorporated fixed effects (ie, aged ≥65 years, sex, comorbidity [ie, none, one, or two or more diseases], one or more COVID-19 vaccinations, SpO_2_ [≥90%] at hospital admission, WHO region, and respiratory support device at hospital admission) and a random effect (facility to account for some unobserved between-facility heterogeneity). The proportional hazards assumption was tested in plots of scaled Schoenfeld residuals for each fixed effect without the random effect term with no observed non-proportionality (p>0·05 for each). Associations were reported as hazard ratios (HRs) with 95% CIs. Statistical significance was defined as a p value of less than 0·05.

The initial sample size target (approximately 125 patients per hospital, up to 4500 patients in total) was set to maximise the precision of descriptive estimates within resource and feasibility constraints. Subsequent formal sample size calculations for the multivariable analyses (including Cox and multistate models), as detailed in the statistical analysis plan, confirmed that the achieved sample size provided sufficient power (≥90%) to detect clinically relevant associations (eg, odds ratio=2) for key transitions and mortality (the primary outcome).

Oxygen support types were categorised as: none; nasal cannula; simple or Venturi face mask; non-rebreather mask with reservoir; HFNO; CPAP; NIV; or invasive mechanical ventilation. Fraction of inspired oxygen (FiO_2_) was calculated from the flow rate and type of oxygen support (appendix p 13), and daily consumption assumed 24 h at the last known flow rate (appendix p 16).

A multistate Markov model was used to evaluate the progression of hospital stays and changes in respiratory support type. In this model, patients were categorised into states corresponding to: standard oxygen therapy (ie, nasal cannula, or face mask with or without reservoir); HFNO, CPAP, or NIV; or invasive mechanical ventilation. Two absorbing states were defined: discharged alive or death. Transition and state occupation probabilities were estimated using the mstate R package.^10^ Proportionality within each transition was visually inspected by Schoenfeld residual plots and showed no major violations, and global tests for proportionality were non-significant.

Entries with absent admission dates were excluded. Missing data were imputed for variables with less than 10% missingness using a non-parametric random forest imputation (missForest R package).^11^ Analyses were performed with R statistical software (version 4.2.3).

### Role of the funding source

The funder of the study had no role in study design, data collection, data analysis, data interpretation, writing of the report, or decision to submit for publication.

## Results

Between Jan 24 and Nov 22, 2022, 53 726 patients were screened and 3070 patients were enrolled (figure 1B). The median age of patients was 63 years (IQR 47–73; data from 3066 patients). 1471 (48·1%) were female and 1590 (51·9%) were male; sex data were not available for nine patients (table 1). COVID-19 was confirmed by rapid diagnostic test or PCR in 878 (28·6%) of 3070. 1683 (54·8%) of 3069 patients had received (ie, before admission) at least one COVID-19 vaccination (table 1). Hypertension was the most common chronic condition (1539 [51·2%] of 3006), followed by diabetes (780 [26·1%] of 2986), and chronic cardiac disease (716 [24·1%] of 2975). Comorbidities were common; 1814 (61·6%) of 2947 patients had two or more underlying chronic conditions. At presentation, most patients were tachypnoeic (median respiratory rate 24 breaths per min [IQR 20–29]), but not tachycardic (median heart rate 90 beats per min [IQR 79–108]; table 1). 1000 (32·6%) of 3066 patients had a reduced level of consciousness (defined as not alert on the AVPU [alert, verbal, pain, unresponsive] scale, or <15 on the Glasgow Coma Scale depending on local protocols).

**Table 1:** Baseline characteristics, patient physiology, and oxygen requirement at hospital admission

		**N**	n (%) or n (IQR)*
Age, years		3066	63 (47–73)
Sex		3061	..
	Male	..	1590 (51·9%)
	Female	..	1471 (48·1%)
BMI, kg/m^2^		2886	..
	Underweight (<18·5)	..	205 (7·1%)
	Normal weight (18·5–24·9)	..	1248 (43·2%)
	Pre-obese (25–29·9)	..	888 (30·8%)
	Obese (≥30)	..	545 (18·9%)
Confirmed pregnancy		1467	15 (1·0%)
Previous COVID-19 vaccination (≥1 doses)		3069	1683 (54·8%)
Comorbidities		2947	..
	0	..	330 (11·2%)
	1	..	803 (27·2%)
	≥2	..	1814 (61·6%)
Specific conditions†			
	Hypertension	3006	1539 (51·2%)
	Diabetes	2986	780 (26·1%)
	Chronic cardiac disease	2975	716 (24·1%)
	Chronic obstructive pulmonary disease	2983	511 (17·1%)
	Current smoking	2896	408 (14·1%)
	Asthma	2984	255 (8·5%)
	Chronic neurological disease	3002	230 (7·7%)
	HIV	2827	198 (7·0%)
	Tuberculosis (current or previous)	2945	189 (6·4%)
	Cancer (current or previous)	2989	158 (5·3%)
Decreased level of consciousness		3066	1000 (32·6%)
Heart rate, beats per min		3066	90 (79–108)
Respiratory rate, breaths per min		2877	24 (20–29)
Systolic blood pressure, mm Hg		3049	128 (110–140)
FiO_2_		2451	40% (28–44)
Oxygen saturation, median %		3061	94% (90–97)
SpO_2_:FiO_2_		2443	243 (196–315)
Oxygen support type		3052	..
	No oxygen (room air)	..	316 (10·4%)
	Nasal cannula	..	1394 (45·7%)
	Simple face mask	..	687 (22·5%)
	Venturi mask	..	67 (2·2%)
	Non-rebreather mask with reservoir	..	265 (8·7%)
	High-flow nasal oxygen	..	49 (1·6%)
	Continuous positive airway pressure	..	38 (1·2%)
	Non-invasive ventilation	..	65 (2·1%)
	Invasive mechanical ventilation	..	171 (5·6%)

Data are median (IQR) or n (%). FiO_2_=fraction of inspired oxygen. SPO_2_=oxygen saturation.

* N indicates the number of patients for whom data were available.

† Only comorbidities occurring in at least 5% of patients are shown.

Patient characteristics varied considerably by region (appendix p 10). Individual disease prevalence varied considerably by region, especially for hypertension (40% in Africa, 79% in Europe) chronic cardiac disease (13% in Africa, 52% in Europe), and diabetes (18% in the Western Pacific, 41% in the Eastern Mediterranean). Notably, patients recruited from African sites were younger than those from other regions, more frequently living with HIV, and more likely to have been previously treated for tuberculosis (appendix pp 10–11).

At the time of enrolment, patients had a median oxygen saturation of 94 (IQR 90–97), with median SpO_2_:FiO_2_ of 243 (IQR 196–315; table 1). Most patients initially received oxygen through nasal cannula (1394 [45·7%] of 3052) or simple face mask (687 [22·5%]; appendix p 12). Only 171 (5·6%) of 3052 patients were on invasive mechanical ventilation at recruitment. Patients from African sites had lower median oxygen saturations (91% [IQR 86–96%]) despite similar median FiO_2_ as the overall study cohort (0·4).

During hospital stay, the highest level of respiratory support remained nasal cannula, or simple or Venturi face masks for most patients (1964 [64·0%] of 3070; appendix p 13). For those receiving oxygen, daily volume requirements remained similar for each day of the first week of admission (appendix p 16). However, volume consumption dropped markedly during the first 4 days among those initially requiring large flow rates through non-rebreather masks or HFNO (appendix p 17). Mechanical ventilation was used in 340 (11·1%) patients overall, with considerable regional variation; 75 (26·4%) of 284 patients received it in the Americas and 95 (17·5%) of 543 patients received it in the Eastern Mediterranean region (appendix p 13). African sites had the highest level of use of non-rebreather reservoir face masks (132 [15·3%] of 861) and the lowest level of HFNO (two [0·2%]), CPAP or NIV (seven [0·8%]), and invasive mechanical ventilation (26 [3·0%]).

HFNO required the most oxygen per day per patient (median 21 144 L per day). Non-rebreather masks were the second highest oxygen-consuming device (median 17 280 L per day; appendix p 18). Patients were treated with oxygen for a median of 6 days (IQR 3–10).

649 (23·4%) of 2779 patients died within the follow-up period, ranging from 53 (10·5%) of 506 in South-East Asia to 286 (37·6%) of 760 in Africa (table 2). Mortality was 8·6% (17/198) in patients who received only low-flow or no oxygen support and 62·9% (205/326) in patients who received invasive mechanical ventilation, with considerable regional variation when stratified by form of oxygen support.

**Table 2:** 30-day mortality overall and by the highest level of oxygen support received during admission, by region

		Overall, n=2779*	Africa, n=760*	Americas, n=284*	Eastern Mediterranean, n=499*	Europe, n=322*	South-East Asia, n=506*	**Western Pacific, n=408**†	**p values**
Length of in-hospital follow-up among survivors, days		7 (4–11)	9 (5–12)	8 (5–13)	10 (5–18)	6 (4–9)	4 (3–7)	10 (7–13)	<0·0001
Outcome (before 31 days)		..	..	..	..	..	..	..	<0·0001
	Dead	649 (23·4%)	286 (37·6%)	68 (23·9%)	116 (23·2%)	53 (16·5%)	53 (10·5%)	73 (17·9%)	..
	Clinically improved	1961 (70·6%)	432 (56·8%)	169 (59·5%)	369 (73·9%)	249 (77·3%)	439 (86·8%)	303 (74·3%)	..
	Not clinically improved	169 (6·1%)	42 (5·5%)	47 (16·5%)	14 (2·8%)	20 (6·2%)	14 (2·8%)	32 (7·8%)	..
Mortality (before 31 days) by highest level of oxygen support									
	No supplemental oxygen	17/198 (8·6%)	15/71 (21·1%)	0/24	1/33 (3·0%)	0/23	0/42	1/5 (20·0%)	0·0001
	Nasal cannula or Venturi or simple masks	268/1766 (15·2%)	197/534 (36·9%)	9/148 (6·1%)	11/260 (4·2%)	6/222 (2·7%)	26/375 (6·9%)	19/227 (8·4%)	<0·0001
	Non-rebreather mask with reservoir	99/258 (38·4%)	58/123 (47·2%)	6/16 (37·5%)	10/36 (27·8%)	1/5 (20·0%)	4/29 (13·8%)	20/49 (40·8%)	0·011
	High-flow nasal oxygen	14/74 (18·9%)	1/2 (50·0%)	2/9 (22·2%)	3/12 (25·0%)	1/8 (12·5%)	5/7 (71·4%)	2/36 (5·6%)	0·0015
	Continuous positive airway pressure or non-invasive ventilation	45/155 (29·0%)	4/5 (80·0%)	4/12 (33·3%)	22/67 (32·8%)	4/17 (23·5%)	10/33 (30·3%)	1/21 (4·8%)	0·016
	Invasive mechanical ventilation	205/326 (62·9%)	10/24 (41·7%)	47/75 (62·7%)	69/90 (76·7%)	41/47 (87·2%)	8/20 (40·0%)	30/70 (42·9%)	<0·0001

Data are median (IQR), n (%), or n/N (%), unless otherwise stated.

* n (%).

† Pearson's Chi-squared test.

**Figure 2:**
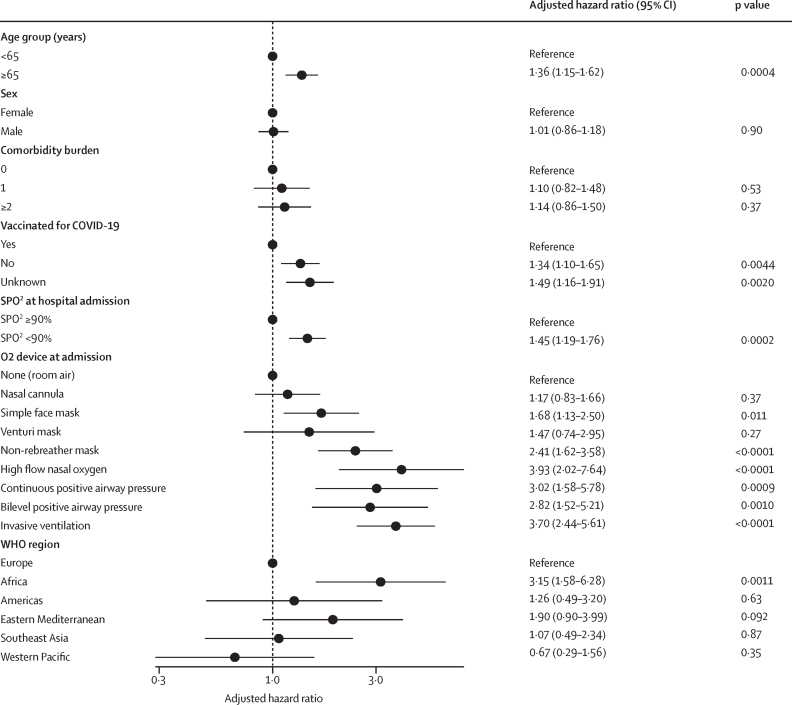
Predictors of inpatient mortality up to 30 days Cox mixed effect model incorporated fixed effects and a random effect (proportion of critical care beds within the facility). Error bars are 95% CIs. SPO_2_=oxygen saturation.

Independent predictors of 30-day hospital mortality included age older than 65 years (HR 1·36 [95% CI 1·15–1·62]; figure 2); no vaccination against COVID-19 (1·34 [1·10–1·65]); baseline SpO_2_ less than 90% (1·45 [1·19–1·76]); and region of recruitment, with patients from Africa showing the highest HR for death compared with those from Europe (3·15 [1·58–6·28]).

Adults aged 65 years or older were significantly more likely to progress from receiving no oxygen or standard oxygen therapy (ie, nasal cannula, or face mask with or without reservoir) to non-invasive respiratory support (adjusted HR 1·43 [95% CI 1·01–2·02]; p=0·045), and to invasive mechanical ventilation (1·80 [1·21–2·70]; p=0·0040; appendix p 19).

Facilities varied widely in their critical care capacity (table 3). As a proportion of overall beds, those used to provide critical care (defined by Marshall and colleagues^12^) were from 3% in Africa and to 33% in the Western Pacific facilities taking part. The median number of maintenance staff per 100 beds was 0·75 (IQR 0·41–1·46), ranging from 0·60 (0·33–0·88) in Africa to 2·31 (1·25–5·50) in the Eastern Mediterranean. Reflecting resource availability and health system configuration, the widest staffing range was seen in total health-care workers per 100 beds (overall median 18 [IQR 6–75], with a median of six [5–11] per 100 in Africa and 110 [28–437] per 100 in the Americas).

**Table 3:** Facility-level descriptions of capacity

		**Overall, n=59**	**Europe, n=6**	**Africa, n=17**	**Americas, n=6**	**Eastern Mediterranean, n=12**	**South-East Asia, n=10**	**Western Pacific, n=8**	p value*
Bed capacity									
	Total	340 (195–700)	170 (109–306)	320 (200–550)	273 (145–576)	365 (150–716)	625 (263–1250)	530 (397–725)	0·25
	Critical care beds proportion	0·16 (0·07–0·39)	0·16 (0·13–0·79)	0·03 (0·02–0·06)	0·21 (0·14–0·47)	0·30 (0·20–0·56)	0·22 (0·10–0·42)	0·33 (0·24–1·00)	<0·0001
Staffing per 100 beds									
	Maintenance staff	0·75 (0·41–1·46)	0·87 (0·65–1·07)	0·60 (0·33–0·88)	1·07 (0·30–1·45)	2·31 (1·25–5·50)	0·37 (0·23–0·53)	0·82 (0·66–1·94)	0·0018
	Health-care workers	18 (6–75)	41 (29–58)	6 (5–11)	110 (28–437)	93 (43–111)	13 (5–20)	17 (14–51)	0·0028
Oxygen devices per 100 beds									
	Ventilators	11 (5–24)	15 (8–28)	3 (2–5)	52 (20–125)	17 (10–23)	10 (5–21)	11 (7–29)	0·0011
	Continuous positive airway pressure or non-invasive ventilation	1·33 (0·55–3·17)	1·31 (0·00–3·10)	0·67 (0·18–1·33)	1·88 (0·19–3·06)	2·90 (1·22–5·90)	2·47 (1·36–3·33)	0·94 (0·54–1·69)	0·024
	High-flow nasal oxygen	1·82 (0·12–4·83)	4·20 (1·09–7·14)	0·00 (0·00–2·00)	8·71 (3·93–18·70)	1·83 (0·61–3·08)	1·98 (0·69–7·53)	3·17 (0·35–11·72)	0·011
Oxygen source									
	Bedside concentrators	38 (64%)	6 (100%)	12 (71%)	3 (50%)	1 (8%)	10 (100%)	6 (75%)	<0·0001
	Cylinders	59 (100%)	6 (100%)	17 (100%)	6 (100%)	12 (100%)	10 (100%)	8 (100%)	..
	Liquid O_2_:pressure swing adsorption plant*	45 (76%)	4 (67%)	8 (47%)	5 (83%)	12 (100%)	10 (100%)	6 (75%)	0·0040
Oxygen delivery									
	Piping	50 (85%)	5 (83%)	12 (71%)	4 (67%)	12 (100%)	10 (100%)	7 (88%)	0·095
	Filling manifold	15 (25%)	1 (17%)	6 (35%)	1 (17%)	1 (8%)	1 (10%)	5 (63%)	0·081
	Distribution manifold	28 (47%)	4 (67%)	5 (29%)	1 (17%)	8 (67%)	7 (70%)	3 (38%)	0·098
Permanent electricity supply		56 (95%)	6 (100%)	14 (82%)	6 (100%)	12 (100%)	10 (100%)	8 (100%)	0·35
Electrical source									
	Generator	48 (81%)	3 (50%)	16 (94%)	2 (33%)	12 (100%)	10 (100%)	5 (63%)	0·0002
	Grid	58 (98%)	6 (100%)	17 (100%)	6 (100%)	12 (100%)	9 (90%)	8 (100%)	0·51
	Solar	13 (22%)	0	5 (29%)	0	4 (33%)	3 (30%)	1 (13%)	0·42

Data are median (IQR) or n (%), unless otherwise stated.

* Kruskal–Wallis rank sum test.

A permanent supply of electricity (24 h per day, 7 days per week) was available to all sites except those in Africa, where 18% of facilities reported interrupted availability (appendix p 20).

Oxygen cylinders were available at all participating sites. However, a facility-level high volume oxygen source (liquid tank or pressure swing adsorption plant) was present in 76% of facilities, with the lowest proportion of sites in Africa (47%). Methods of oxygen delivery and transport within the facility showed similar heterogeneity (appendix p 21).

## Discussion

This large international study describes a large cohort of patients admitted to hospital with suspected or confirmed COVID-19 and respiratory distress, across 23 LMICs. There was considerable intra-regional variation in types of respiratory support provided to patients, which was also reflected in the availability of oxygen delivery methods. For example, in African facilities, lower volume oxygen sources were more common, and there were fewer devices to provide invasive mechanical and non-invasive ventilation and high-flow nasal oxygen therapy. Infrastructural limitations were evident in regions more reliant on locally generated electricity and with inconsistent grid supplies. Consistent with this observation, a number of patients with hypoxaemia on admission were not placed on oxygen support, presumably due to little capacity. In this subgroup, mortality in Africa was 21%, compared with 2% in all other regions combined.

The results suggest that variations in mortality across regions could be explained at least partly by oxygen supply and device use. Organisational factors are also likely to contribute to patient outcomes. Health-care worker availability was lowest in Africa (median six per 100 beds *vs* 18 per 100 overall), as was the proportion of critical care beds available (3% *vs* 16% overall). However, there was no direct correlation between mortality and staffing resources by region: the South-East Asia region had the lowest mortality (10%) compared to all other regions, and also fewer health-care workers (13 per 100 beds) than average. We did not perform a facility-level outcome analysis.

For patients with severe acute respiratory infection whose hypoxaemia persists despite low-flow oxygen with passive interfaces, escalation of care has typically involved invasive mechanical ventilation. In our study, the overall mortality rate among patients requiring ventilation was 63%. The highest mortality among ventilated patients was in Europe (87%, although only 14% of patients received ventilation). By contrast, mortality among ventilated patients was lower in Africa (42%) and South-East Asia (40%), where a much smaller proportion of patients received ventilation. We hypothesise that this finding is explained by less strict patient selection with a higher availability of ventilation, resulting in the treatment of patients with poorer prognosis. Early experience in the pandemic reflects this pattern in high-income countries. In parallel, recognition of resource inequity resulted in the widespread provision of ventilators, many of which were simplified devices intended for low-resource settings, and in the increased use of non-invasive respiratory support, such as high-flow nasal oxygen. Notably, the RECOVERY-RS trial showed a reduction in intubation afforded by the early use of CPAP for acute hypoxaemic respiratory failure.^13^ Both technologies were available before the pandemic, but our study shows inequity in global provision. Reliance on non-rebreather masks led to the second highest oxygen volume requirement, consistent with other studies,^14^ in addition to a very high observed mortality. In our adult population, we found that HFNO used the most oxygen per patient-day. However, the potential for strict titration HFNO protocols to reduce this consumption has been shown in a randomised controlled trial in children, which optimised air flow.^15^ Provision of earlier alternative oxygen interventions, such as NIV, could also bring advantages in protecting the limited volume of medical oxygen and improving outcomes.

Notably, provision of respiratory support devices alone, without the necessary infrastructure and clinical support, is unlikely to improve patient survival.^16^ A complex ecosystem of support including biomedical and health-care professionals is required to maintain equipment function, training, and quality of care. The investment in the Eastern Mediterranean region in biomedical engineer support, with staff per bed ratios, is more than double any other region. Elsewhere, our study showed heterogeneity in both the numbers of maintenance staff and the number of ventilators available across regions, with some regions having higher values for both; however, a formal correlation between these variables was not assessed. A large number of pertinent clinical questions remain unanswered with respect to oxygen support for patients with COVID-19, and more broadly for patients with severe acute respiratory infections; the optimal timing, triggers for escalation, and patient selection are undefined, particularly in LMICs. Improving the performance and calibration of pulse oximeters for patients with darker skin pigmentation is similarly urgent,^17^ although we were unable to address this issue within our study design.

Outside of the direct effects of provisions of health care, important determinants of patient outcomes exist at the population level, and geographical variation has been well reported in COVID-19 and severe acute respiratory infection.^18^ Our study shows rates of multiple comorbidity in excess of 50% in all regions, and more nuanced regional differences in underlying individual diseases, which are well aligned with previous findings (eg, a South African cohort).^19^ Every regional cohort had rates of hypertension in excess of 40%, and of diabetes exceeding 18%. The global effects of chronic disease on susceptibility to severe respiratory infection should lead to even more support for public health strengthening to address them. The effect of HIV as the primary global cause of immunosuppression remains stark.

Our study provides data at both the patient and facility levels, allowing insights into how infrastructural availability and clinical decisions are related. The size and prospective and consecutive recruitment allowed the minimisation of bias and reflect real-world patient populations with respect to outcomes and risk factors, particularly when randomised controlled trials were not running. The inclusion of sites with very limited or no previous research experience enabled capacity building, enhanced visibility of overlooked sites, and the embedding of research into everyday clinical care.

As this was a cohort study, causal inferences cannot be confidently drawn. We note also the inherent simplification of geographical classification that overlooks substantial within-region variability, and the limitations imposed by sample size, which affect power to understand subregional patterns. The sites included within the study might not be fully representative of facilities in respective countries, nor of whole regions, due to the research governance stipulations we imposed as site selection criteria. To improve facility representation and encourage research capacity building, we were, in most cases, able to include pairs of hospitals that represented one academic or tertiary-level hospital and one community or district-level hospital.

Given the specific timeframe of the COVID-19 pandemic during which our data collection occurred, this study likely represents disease dominated by omicron variants. Sequencing of SARS-CoV-2 strains was not available at most sites. Furthermore, changes in hospitalisation patterns and reductions in case-fatality ratio during the study period might limit the generalisability of our findings to subsequent viral strains. The competing demands between research and health-care delivery also resulted in limited data availability. For example, universal COVID-19 testing and more broad aetiological investigation were not possible. However, these real-world limitations, and the scarcity of access to diagnostics more broadly, underscore the ongoing inequity even outside of health emergencies.

We strongly welcome the major recent shift towards high-level advocacy and the development of oxygen ecosystems, including the *Lancet Global Health* Oxygen Commission^2^ and the Global Oxygen Alliance.^20^ Globally enhanced medical oxygen provision will benefit patients requiring emergency, critical, and operative care, and support robust pandemic preparedness planning. Scaling oxygen capacity will need strong focus outside of tertiary centres, where the inequity is most stark.^21^ In parallel with this expansion in medical oxygen availability, additional practical clinical studies are also needed to examine clinical questions of special relevance in LMICs. Some notable trials are underway (ISRCTN15622505, NCT05754034, and NCT04693403) or have been recently reported,^15^, ^22^, ^23^ but outstanding priorities include understanding which patients will benefit from novel delivery protocols, how to identify hypoxaemia accurately irrespective of skin pigmentation, and how to implement scalable programmes for support of patients admitted to hospital with severe acute respiratory infection.

Patients deserve a concerted and broad coalition of partners to meet these needs. WHO is committed to collecting and using high quality trial evidence to improve patient outcomes, and has established a collaboration of researchers and institutions working towards global pragmatic (platform) trials;^24^, ^25^ we encourage interested clinicians, institutions, and networks to join us.^26^

### Contributors

### Data sharing

All raw and processed datasets supporting this study are openly available online. Data analysis and processing code are publicly accessible on GitHub.

## Declaration of interests

We declare no competing interests.
