# Supplementary material for: Medical oxygen and respiratory support requirements for patients hospitalised with COVID-19 in 23 low-income and middle-income countries: a prospective, observational cohort study
Source: Lancet Glob Health. 2026 Jul 1;14(2):e233–41. doi: 10.1016/S2214-109X(25)00480-2 (PMC12795722; doi:10.1016/S2214-109X(25)00480-2)

THE LANCET
Global Health

Supplementary appendix

This appendix formed part of the original submission and has been peer reviewed. We post it as supplied by the authors.

Supplement to: Relan P, Rylance J, Arabi YM, et al. Medical oxygen and respiratory support requirements for patients hospitalised with COVID-19 in 23 low-income and middle-income countries: a prospective, observational cohort study. *Lancet Glob Health* 2026; **14:** e233–41.

Supplemental material

Full author list for PubMed listing 1
Full author contribution list 6
Acknowledgements 8
Supplemental data 9

Supplementary table 1. Covariates which had missing data which were singly imputed using random forest 9
Supplementary table 2. Baseline characteristics and oxygen support modality by region 10
Supplementary table 3. Respiratory support at time of enrolment 12
Supplementary table 4. Length of hospital stay and highest level of oxygen support by region 13
Supplementary table 5. Basis of calculations of FiO_2 13
Supplementary table 6. Summary of ethical approvals for the study 14
Supplementary table 7. Unadjusted hazard ratios (HR) for the mode of oxygen delivery and region 15
Supplementary figure 1. Distribution of oxygen requirement per patient during first seven days of admission 16
Supplementary figure 2. Distribution of daily oxygen per day of follow up stratified by oxygen delivery mode at admission 17
Supplementary figure 3. Daily oxygen consumption by type of respiratory support 18
Supplementary figure 4. Trajectory of oxygen support levels and associated outcomes 19
Supplementary figure 5. Electricity source by region 20
Supplementary figure 6. Oxygen source by region (radar plot) 21
Supplementary figure 7. Trajectory of oxygen support modality and associated outcomes 22

Full author list for PubMed listing

Name for citing	Institution	Highest degree	ORCID
**Pryanka Relan**	World Health Organization, Geneva, Switzerland	MD	0000-0002-9543-7891
**Jamie Rylance**	World Health Organization, Geneva, Switzerland	PhD	0000-0002-2323-3611
**Yaseen Arabi**	King Saud bin Abdulaziz University for Health Sciences, Riyadh, Kingdom of Saudi Arabia	MD	0000-0001-5735-6241
**Pauline Convocar**	Southern Philippines Medical Center, Davao City, Philippines	MD	
**Matthieu Rolland**	World Health Organization, Geneva, Switzerland	PhD	
**Janet Diaz**	World Health Organization, Geneva, Switzerland	MD	
**Islam Gamal Albayadi**	Suez Canal University, Ismaïlia, Egypt	MD	
**Ahmad Al-Touny**	Suez Canal University, Ismaïlia, Egypt	MD	
**Aiman Al-Touny**	Suez Canal University, Ismaïlia, Egypt	MD	0000-0003-2713-1281
**Shimaa Ahmed Hamed Al-Touny**	Suez Canal University, Ismaïlia, Egypt	MD	0000-0001-5816-2799
**Carlos Arturo Alvarez-Moreno**	Universidad Nacional de Colombia, Bogota, Colombia	MD	0000-0001-5419-4494
**Gasim Amrahli**	Regional Medical Divisions Administration, Azerbaijan	MD	
**Djillali Annane**	Raymond Poincaré University Hospital (AP-HP), Paris, France	PhD	0000-0001-6805-8944
**Zeina Aoun Bacha**	Saint Joseph University, Beirut, Lebanon	MD	0000-0001-9723-1267
**John Adabie Appiah**	World Health Organization, Geneva, Switzerland	MD	0000-0002-0386-6120
**Masooma Aqeel**	Aga Khan University, Karachi, Pakistan	MD	0000-0001-5276-7017
**Diptesh Aryal**	Nepal Intensive Care Research Foundation, Kathmandu, Nepal	PhD	
**Hope Atuhaire**	Walimu, Kampala, Uganda	MD	
**Celine Baaklini**	Saint Joseph University, Beirut, Lebanon	MD	0009-0005-8860-8858
**Barnabas Bakamutumaho**	Uganda Virus Research Institute, Kampala, Uganda	MD	
**Debashis Basu**	University of Pretoria, Pretoria, South Africa	MD	
**Neale Batra**	World Health Organization, Geneva, Switzerland	MD	0000-0003-4361-681X
**Abigail Beane**	Mahidol Oxford Tropical Medicine Research Unit, Thailand	PhD	0000-0001-7046-1580
**Katrina Bentulan**	Southern Philippines Medical Center, Davao City, Philippines	RN	
**Benilde Bepouka**	University of Kinshasa, Kinshasa, Democratic Republic of the Congo	PhD	0000-0003-3063-4234
**Anil Bilimale**	Jagadguru Sri Shivarathreeshwara University, Mysore, India	MD	0000-0002-3601-2930
**Nguyen Thien Binh**	Trung Vuong Hospital, Ho Chi Minh City, Vietnam	MD	
**Kieran Bligh**	UNICEF, Washington DC, United States of America	MPH	

**Ina Bolocan**	Ministry of Health, Moldova	MD	
**Guillermo Caceres-Cardenas**	Universidad Peruana Cayetano Heredia, Lima, Peru	MD	0000-0003-1447-5608
**Luis Alberto Camputaro**	Universidad de Buenos Aires, Buenos Aires, Brazil	PhD	0000-0003-0291-0190
**Itziar Carrasco-Garcia**	World Health Organization, Geneva, Switzerland	MD	0000-0002-9974-3378
**Devasahayam J Christopher**	Christian Medical College, Vellore, India	MD	0000-0002-9405-8494
**Mônica Cruz**	Fundação Oswaldo Cruz, Rio de Janeiro, Brazil	MD	0000-0003-0641-0097
**Matthew Cummings**	Columbia University, New York, United States	MD	
**Shaimaa Dahshan**	Suez Canal University, Ismaïlia, Egypt	MD	
**Ichinnorov Dashtseren**	Mongolian National University of Medical Sciences, Ulaanbaatar, Mongolia	PhD	0000-0001-6413-4794
**Cinzia De Brito Procopio**	World Health Organization, Geneva, Switzerland		
**Mohammed Derow**	Ministry of Health and Human Service, Mogadishu, Somalia	MD	
**Jaidev Devadas**	Father Muller Medical College, Mangalore, India	MD	0000-0003-3332-7766
**Sara Domínguez-Rodríguez**	Universidad Europea, Madrid, Spain	PhD	0000-0003-3747-9265
**Alejandro Jose Duarte Cuellar**	Ministry of Health of El Salvador, San Salvador, El Salvador	MD	0009-0002-6925-5266
**Ryenchindorj Erkhembayar**	Mongolian National University of Medical Sciences, Ulaanbaatar, Mongolia	MD	0000-0002-0603-184X
**Mario Fernando Escobar**	Colsanitas, Bogotá ,Colombia	MD	
**Adeniyi Francis Fagbamigbe**	University of Ibadan, Ibadan, Nigeria	PhD	0000-0001-9184-8258
**Faith Joan Gaerlan**	Southern Philippines Medical Center, Davao City, Philippines	MD	0009-0001-9357-9343
**Aniruddha Ghose**	Chittagong Medical College, Chattogram, Bangladesh	MD	0000-0002-5551-6691
**Delmy Virginia Granados Castro**	Hospital Nacional Saldaña, San Salvador, El Salvador	MD	0000-0002-2874-3514
**Bridget Griffith**	Clinton Health Access Initiative, Boston, United States of America	PhD	0000-0003-4804-7919
**Christophe Guitton**	World Health Organization, Geneva, Switzerland		
**Nicole Haber**	Bellevue Medical Center, Mansourieh, Lebanon	MD	
**Bassem Habr**	Hotel Dieu de France University Hospital, Beirut, Lebanon	MD	
**Priscilla Haguma**	Walimu, Kampala, Uganda	PhD	0000-0002-1690-9070
**Rashan Haniffa**	Mahidol Oxford Tropical Medicine Research Unit, Bangkok, Thailand	PhD	0000-0002-8288-449X
**Madiha Hashmi**	Ziauddin University, Karachi, Pakistan	MD	0000-0002-7332-0692

**Than Manh Hung**	National Hospital of Tropical Diseases, Hanoi, Vietnam	MD	0000-0002-3227-6947
**Shevin T Jacob**	Liverpool School of Tropical Medicine, Liverpool, UK	MD	0000-0003-2425-9394
**Rashidatu Fouad Kamara**	World Health Organization Regional Office for Africa, Brazzaville, Congo	MD	0000-0002-3787-8752
**Leticia Kawano**	Universidade de Sao Paulo, Sao Paulo, Brazil	PhD	0000-0003-0784-1331
**Shirish KC**	World Health Organization, Geneva, Switzerland	MD	
**Muhammad Haroon Khan**	Aga Khan University, Karachi, Pakistan	MD	
**Zohair Ahmed Khan**	Aga Khan University, Karachi, Pakistan	MD	0000-0001-6672-3111
**Basheer Khassawneh**	Jordan University of Science and Technology, Irbid, Jordan	MD	0000-0003-2160-4498
**Khalid Kheirallah**	Jordan University of Science and Technology, Irbid, Jordan	PhD	0000-0003-4504-4472
**Francis Kiweewa**	Lira Regional Referral Hospital, Lira, Uganda	MD	0000-0003-4938-9558
**Mark Kizito**	Kiruddu National Referral Hospital, Kampala, Uganda	MD	0000-0003-1906-3442
**Chiori Kodama**	World Health Organization Regional Office for the Eastern Mediterranean, Cairo, Egypt	MD	
**Chamira Kodippily**	Network for Improving Critical Care Systems and Training, Colombo, Sri Lanka	BSc	
**Richard Kojan**	Alima, Paris, France	MD	0000-0003-3415-2830
**Ashok Kumar**	Dr. Ziauddin Hospital, Karachi, Pakistan	MD	0000-0003-4685-2631
**Gary Kuniyoshi**	World Health Organization Regional Office for the Eastern Mediterranean, Cairo, Egypt	MD	
**Arthur Kwizera**	Makerere University College of Health Sciences, Kampala, Uganda	MD	0000-0002-7025-0465
**Ingrid Lara**	World Health Organization, Geneva, Switzerland	MSc	
**Juan Carlos Llontop Otero**	Hospital de Huaycán, Huaycán, Peru	MD	
**Nombulelo Magula**	University of KwaZulu Natal, Durban, South Africa	PhD	0000-0001-8625-9539
**Ata Mahmoodpoor**	Tabriz University of Medical Sciences, Tabriz, Iran	MD	0000-0002-4361-6230
**Jean Robert Makulo**	Université de Kinshasa, Kinshasa, Democratic Republic of the Congo	PhD	0000-0001-5517-3281
**Undram Mandakh**	Mongolian National University of Medical Sciences, Ulaanbaatar, Mongolia	MD	0000-0001-9624-164X
**John C Marshall**	University of Toronto, Toronto, Canada	MD	0000-0002-7902-6291
**Mohlamme John Mathabathe**	Steve Biko Academic Hospital, Pretoria, South Africa	MD	0000-0002-1276-752X
**Rabiul Alam Md Erfan Uddin**	Chittagong Medical College, Chattogram, Bangladesh	MD	0000-0002-9161-0175
**Carlos Medina**	Hospital de Huaycán, Huaycán, Peru	MD	
**Maria Mendes**	Independent, Guinea-Bissau	RN	
**Martin Meremikwu**	University of Calabar, Calabar, Nigeria	MD	0000-0002-6144-9413

**Deebya Raj Mishra**	B P Koirala Institute of Health Sciences, Dharan, Nepal	MD	0000-0003-1419-3803
**Srinivas Murthy**	University of British Columbia, Vancouver, Canada	MD	0000-0002-9476-839X
**Kenneth Doya Nonesa**	Southern Philippines Medical Center, Davao City, Philippines	MD	
**Sharon Nyesiga**	Walimu, Kampala, Uganda	MD	0000-0003-0009-5030
**Mulinda Nyirenda**	Kamuzu University of Health Sciences, Blantyre, Malawi	MD	0000-0003-2037-7449
**Chimedsuren Ochir**	Mongolian National University of Medical Sciences, Ulaanbaatar, Mongolia	PhD	0000-0002-7134-5255
**Alina Ogizbayeva**	Karaganda Medical University, Karaganda, Kazakhstan	PhD	0000-0003-1006-1870
**Adejumobi Oluwabukola**	World Health Organization, Ogun State, Nigeria	MPH	
**Bernard Omech**	Lira University, Lira, Uganda	MD	0000-0002-3087-736X
**Darius Owachi**	Kiruddu National Referral Hospital, Kampala, Uganda	MD	0000-0002-4785-1887
**Parash Pandey**	B.P. Koirala Institute of Health Sciences, Kathmandu, Nepal	MD	0009-0005-4834-0332
**Luigi Pisani**	World Health Organization, Geneva, Switzerland	MD	
**Sanjeev Rai**	Father Muller Medical College Hospital, Mangaluru, India	MD	0000-0002-8692-9050
**Gayle Alyannah A. Ramos-Lee**	Lung Center of the Philippines, Manila, Philippines	MD	
**Sumayyah Rashan**	National Intensive Care Surveillance - MORU (NICS-MORU), Colombo, Sri Lanka	MD	0000-0001-8657-9639
**Bassma Raslan**	Suez Canal University, Ismaïlia, Egypt	MD	
**Ludovic Reveiz**	Pan American Health Organization, Washington DC, United States of America	PhD	0000-0003-3186-9174
**Carlos Alfonso Reyes Silva**	El Salvador Hospital, San Salvador, El Salvador	MD	
**Rozelyn D Reyes-Mauro**	Southern Philippines Medical Center, Davao City, Philippines	MD	
**Moussa Riachi**	Hotel Dieu de France University Hospital, Beirut, Lebanon	MD	0000-0003-2146-6945
**Elisabeth Riviello**	Beth Israel Deaconess Medical Center, Boston, United States of America	MD	0000-0002-9443-3928
**Jose Antonio Rojas Gambasica**	Colsanitas, Bogotá ,Colombia	MD	0000-0002-7489-0187
**Sairah Sadaf**	Sheikh Zayed Medical College, Punjab, Pakistan	MD	
**Maximiliano Ivan Sánchez**	Instituto Especializado Hospital, San Salvador, El Salvador	BSc	
**Amadou Seck**	Independent, Senegal	MSc	
**Kiran Shetty**	Father Muller Medical College Hospital, Mangaluru, India	MD	0009-0003-4220-188X
**Howard Sobel**	World Health Organization Regional Office for the Western Pacific, Manila, Philippines	PhD	0000-0002-1684-0499
**Hassan Soleimanpour**	Tabriz University of Medical Sciences, Tabriz, Iran	MD	0000-0002-1311-4096
**Elizabeth Stanway**	World Health Organization, Geneva, Switzerland	MD	
**Yedilbayeva Tanzira**	Karaganda State Medical University, Karaganda, Kazakhsan	MD	0000-0001-8928-2400
**Eman Teema**	Suez Canal University, Ismaïlia, Egypt	MD	0000-0003-2365-4544

**Dennis Teo**	Lung Center of the Philippines, Manila, Philippines	MD	0009-0009-0239-8212
**Louise Thwaites**	Oxford University Clinical Research Unit, Ho Chi Minh City, Vietnam	PhD	0000-0002-4666-9813
**Le Mau Toan**	Hospital for Tropical Diseases, Ho Chi Minh City, Vietnam	MD	
**Khanyisile Tshabalala**	University of Pretoria, Pretoria, South Africa	MD	0000-0002-6018-7212
**Cesar Ugarte-Gil**	Universidad Peruana Cayetano Heredia, Lima, Peru	PhD	0000-0002-2833-9087
**Benedict Edward Valdez**	Southern Philippines Medical Center, Davao City, Philippines	MD	
**Laura Alejandra Velez Ruiz Gaitan**	World Health Organization, Geneva, Switzerland	MSc	0009-0009-2724-3276
**Valdilea G. Veloso**	Fundação Oswaldo Cruz, Rio de Janeiro, Brazil	PhD	0000-0002-6622-3165
**Katerine Milagros Villaizan Paliza**	Universidad Peruana Cayetano Heredia, Lima, Peru	MSc	0000-0001-5810-1371
**Julie Viry**	World Health Organization, Geneva, Switzerland	MPP	
**Wangari Waweru**	Aga Khan University Hospital, Nairobi, Kenya	PhD	
**Pushpa Ranjan Wijesinghe**	World Health Organization Regional Office for South-East Asia, New Delhi, India	MD	0000-0002-7875-0165
**Prashanth YM**	Father Muller Medical College Hospital, Mangaluru, India	MD	0000-0003-2428-4434
**Marija Zdravkovic**	University Hospital Medical Center Bezanijska kosa, Belgrade, Serbia	PhD	0000-0003-4059-0263
**Milena Zivanovic**	University Hospital Medical Center Bezanijska kosa, Belgrade, Serbia	MD	0000-0003-3436-6863

Full author contribution list

Conceptualization: Djillali Annane, Pauline Convocar, Janet Diaz, Sara Domínguez-Rodríguez, John C Marshall, Srinivas Murthy, Pryanka Relan, Matthieu Rolland, Wangari Waweru

Data curation: Islam Gamal Albayadi, Ahmad Al-Touny, Aiman Al-Touny, Shimaa Ahmed Hamed Al-Touny, Zeina Aoun Bacha, Abigail Beane, Anil Bilimale, Itziar Carrasco-Garcia, Mônica Cruz, Sara Domínguez-Rodríguez, Ryenchindorj Erkhembayar, Mario Fernando Escobar, Adeniyi Francis Fagbamigbe, Faith Joan Gaerlan, Rashan Haniffa, Shevin T Jacob, Leticia Kawano, Shirish KC, Basheer Khassawneh, Khalid Kheirallah, Francis Kiweewa, Mark Kizito, Chamira Kodippily, Ashok Kumar, John Mathabathe, Martin Meremikwu, Deebya Raj Mishra, Kenneth Doya Nonesa, Sharon Nyesiga, Mulinda Nyirenda, Alina Ogizbayeva, Adejumobi Oluwabukola, Bernard Omech, Darius Owachi, Parash Pandey, Luigi Pisani, Sanjeev Rai, Gayle Alyannah A. Ramos-Lee, Sumayyah Rashan, Bassma Raslan, Pryanka Relan, Jamie Rylance, Maximiliano Ivan Sánchez, Amadou Seck, Yedilbayeva Tanzira, Eman Teema, Dennis Teo, Louise Thwaites, Khanyisile Tshabalala, Cesar Ugarte-Gil, Katerine Milagros Villaizan Paliza, Marija Zdravkovic, Milena Zivanovic

Resources: Kieran Bligh, Itziar Carrasco-Garcia, Cinzia De Brito Procopio, Sara Domínguez-Rodríguez, Bridget Griffith, Christophe Guitton, Ingrid Lara, Elizabeth Stanway, Laura Alejandra Velez Ruiz Gaitan

Access and verification of underlying data: Pryanka Relan, Matthieu Rolland, Jamie Rylance

Formal analysis: Yaseen Arabi, Pauline Convocar, Janet Diaz, Pryanka Relan, Matthieu Rolland, Jamie Rylance

Investigation: Islam Gamal Albayadi, Ahmad Al-Touny, Aiman Al-Touny, Shimaa Ahmed Hamed Al-Touny, Carlos Arturo Alvarez-Moreno, Gasim Amrahli, Zeina Aoun Bacha, Hope Atuhaire, Celine Baaklini, Barnabas Bakamutumaho, Debashis Basu, Katrina Bentulan, Benilde Bepouka, Anil Bilimale, Guillermo Caceres-Cardenas, Mônica Cruz, Shaimaa Dahshan, Ichinnorov Dashtseren, Jaidev Devadas, Ryenchindorj Erkhembayar, Adeniyi Francis Fagbamigbe, Faith Joan Gaerlan, Aniruddha Ghose, Priscilla Haguma, Than Manh Hung, Shevin T Jacob, Basheer Khassawneh, Khalid Kheirallah, Francis Kiweewa, Mark Kizito, Juan Carlos Llontop Otero, Nombulelo Magula, Ata Mahmoodpoor, Jean Robert Makulo, John Mathabathe, Rabiul Alam Md Erfan Uddin, Carlos Medina, Martin Meremikwu, Deebya Raj Mishra, Mulinda Nyirenda, Chimedsuren Ochir, Alina Ogizbayeva, Adejumobi Oluwabukola, Darius Owachi, Parash Pandey, Gayle Alyannah A. Ramos-Lee, Bassma Raslan, Carlos Alfonso Reyes Silva, Rozelyn D Reyes-Mauro, Jose Antonio Rojas Gambasica, Jamie Rylance, Eman Teema, Dennis Teo, Louise Thwaites, Khanyisile Tshabalala, Cesar Ugarte-Gil, Benedict Edward Valdez, Valdilea G. Veloso, Katerine Milagros Villaizan Paliza, Marija Zdravkovic

Methodology: Gasim Amrahli, Djillali Annane, Yaseen Arabi, Diptesh Aryal Devasahayam J Christopher, Pauline Convocar, Mohammed Derow, Janet Diaz, Sara Domínguez-Rodríguez, Madiha Hashmi, Leticia Kawano, Richard Kojan, Arthur Kwizera, John C Marshall, Maria Mendes, Srinivas Murthy, Pryanka Relan, Elisabeth Riviello, Matthieu Rolland, Wangari Waweru

Project administration: Aiman Al-Touny, Shimaa Ahmed Hamed Al-Touny, Carlos Arturo Alvarez-Moreno, Zeina Aoun Bacha, Masooma Aqeel, Neale Batra, Anil Bilimale, Nguyen Thien Binh, Ina Bolocan, Mônica Cruz, Alejandro Jose Duarte Cuellar, Faith Joan Gaerlan, Nicole Haber, Madiha Hashmi, Shevin T Jacob, Muhammad Haroon Khan, Khalid Kheirallah, Francis Kiweewa, Undram Mandakh, Martin Meremikwu, Deebya Raj Mishra, Mulinda Nyirenda, Adejumobi Oluwabukola, Sanjeev Rai, Pryanka Relan, Jose Antonio Rojas Gambasica, Jamie Rylance, Amadou Seck, Eman Teema, Louise Thwaites, Le Mau Toan, Khanyisile Tshabalala, Valdilea G. Veloso, Julie Viry

Supervision: Aiman Al-Touny, Carlos Arturo Alvarez-Moreno, Gasim Amrahli, Zeina Aoun Bacha, John Adabie Appiah, Masooma Aqeel, Yaseen Arabi, Diptesh Aryal, Celine Baaklini, Barnabas Bakamutumaho,

Abigail Beane, Anil Bilimale, Nguyen Thien Binh, Ina Bolocan, Luis Alberto Camputaro, Devasahayam J Christopher, Pauline Convocar, Mônica Cruz, Matthew Cummings, Ichinnorov Dashtseren, Mohammed Derow, Jaidev Devadas, Adeniyi Francis Fagbamigbe, Faith Joan Gaerlan, Aniruddha Ghose, Delmy Virginia Granados Castro, Nicole Haber, Bassem Habr, Rashan Haniffa, Madiha Hashmi, Shevin T Jacob, Rashidatu Fouad Kamara, Leticia Kawano, Shirish KC, Zohair Ahmed Khan, Basheer Khassawneh, Khalid Kheirallah, Francis Kiweewa, Chiori Kodama, Chamira Kodippily, Richard Kojan, Ashok Kumar, Gary Kuniyoshi, Arthur Kwizera, Nombulelo Magula, Jean Robert Makulo, Undram Mandakh, Maria Mendes, Martin Meremikwu, Deebya Raj Mishra, Mulinda Nyirenda, Chimedsuren Ochir, Alina Ogizbayeva, Adejumobi Oluwabukola, Bernard Omech, Luigi Pisani, Sumayyah Rashan, Pryanka Relan, Ludovic Reveiz, Moussa Riachi, Elisabeth Riviello, Jose Antonio Rojas Gambasica, Jamie Rylance, Sairah Sadaf, Kiran Shetty, Howard Sobel, Hassan Soleimanpour, Louise Thwaites, Le Mau Toan, Khanyisile Tshabalala, Cesar Ugarte-Gil, Valdilea G. Veloso, Wangari Waweru, Pushpa Ranjan Wijesinghe, Prashanth YM

Writing – original draft: Yaseen Arabi, Pauline Convocar, Janet Diaz, Pryanka Relan, Matthieu Rolland, Jamie Rylance

Writing – review & editing: All authors.

Acknowledgements
This work would not have been possible without the support of a great number of people, who we thank for their time and expertise. In particular, our thanks to all the patients and their carers, and to all the study staff who made the study a success. We apologise that we have been unable to name everyone below.

Study site support, including: Gangavarapu Aasweeja, Bode Abraka, Sotayo Oluwakemi Adenike, Ememobong Aquaisua, Iwara Arikpo, Maria Bahramian, Sulochanadevi B Chakrashali, Moses Bernard, Nimisha Bhatu, Anathi Bonisani, Christian Braga, Zahra Cheema, Henna Dhar, Oluwabunmi Motunrayo Fatungase, Stela Gheorghita, Chandana G Gowda, HN Chandana, Vo Tan Hoang, Tiberiu Holban, HP Poorvitha, Muhammad Muneeb Hussain, Ehimario Uche Igumbor, Zahid Iqbal, Midhuna Iype, Anila Jasmine, Alena Joseph, Sylvester Kaimba, Sai Sri Krishnaja Kanakaveti, Akanksha B Karande, Julius Katungye, Maureen Kayaga, KB Accamma, KB Chethak, KC Harithanath, Quratulain Khan, Lubna KU Jesin, Sanika Ratnakar Kulkarni, Rituparna Kundu, Rose Kurian, R Lathika, Chelsea Lipoto, M Raghavi, Florence Malowa, MD Darshan, Maximciuc Mirabela, Zanele Mlambisa, Negar Mokabber, MP Primisha, Neha Muhammed, Maria Nantaayi, Lynn Nattabi, Deborah Ndukwu, Trung Truong Ngoc, Huynh Ky Nguyen, Fathima Nidha, K Nimithamohan, Immaculate Nuwahereza, Saulos Nyirenda, Elizabeth Ogar, Shotayo Olukemi, Odey Lydia Omgbonya, ON Sreedevi, Deborah Osisanwo, P Ashok, Urvi Patel, Pavel Micsanschi, Thanh Pham Ba, Tabea Phashe, Jacob Phulusa, Valentina Poting-Rascov, Gunisetty Hari Prakash, Ana Quintana, R Nisarga, Ghulam Rasheed, Abid Rasool, Minu Maria Rose, Rotari Ruslan, Arsha Royal, Irina Russu, Daisy G Sampreetha, Nicholus Sebudde, SG Likitha, Zahra Sheikhalipour, Shantanu Shrivastava, Andani Singo, Mounika M Sree, Majid Tagizadeh, Chetana C Tavaragundi, Esther Tayo, Ramapani Thammali, Kieu Pham Tieu, TM Amulya, Opar Bernard Toliva, Kátia Torres, Ubong Udoh, Aparna Uthaman, V Davana, Wesley van Hougenhouck-Tulleken, Harini Vasudev, Nosipho Vilakazi, C Vishwaprasad, Deepika Yadav, Lam Minh Yen, Hakeem Abiola Yusuff.

Study steering committee for providing oversight of the methodology and conduct of the study: Gasim Amrahli, Yaseen Arabi, Diptesh Aryal, DJ Christopher, Pauline Convocar, Mohammed Derow, Sara Dominguez Rodriguez, Madiha Hashmi, Leticia Kawano-Dourado, Richard Kojan, Arthur Kwizera, Ingrid Lara Rendon, John Marshall, Maria Mendes, Srin Murthy, Beth Riviello, Wangari Waweru-Siika.

WHO study support team, including clinical management focal points from all WHO regions: John Appiah, Neal Batra, Kieran Bligh, Itziar Carrasco, Cinzia De Brito Procopio, Janet Diaz, Sara Dominguez Rodriguez, Bridget Griffith, Christophe Guitton, Rashidatu Kamara, Chiori Kodama, Gary Kuniyoshi, Dina Pfeifer, Pryanka Relan, Ludovic Reveiz, Matthieu Rolland, Amadou Seck, Howard Sobel, Elizabeth Stanway, Joana Vasques, Ale Velez, Julie Viry, Pushpa Wijesinghe.

Respiratory support research working group for their contributions to the research roadmap for COVID-19 prioritisation: Neill Adhikari, Djillali Annane, Diptesh Aryal, Tim Baker, Itziar Carrasco, Sylvie Chevret, Devasahayam J Christopher, Janet Diaz, Sara Dominguez Rodriguez, Rob Fowler, Martha Gartley, Ewan Goligher, Bridget Griffith, Richard H Kallet, Rashan Haniffa, Devachandran Jayakumar, Richard Kojan, Arthur Kwizera, Ingrid Laura, Jie Li, John Marshall, Bruno Martins Tomazini, Armand Mekontso-Dessap, Srinivas Murthy, Christian Paletta, Pryanka Relan, Elisabeth Riviello, Jorge Salluh, Jonathan AC Sterne, Bharath Kumar Tirupakuzhi Vijayaraghavan, Wangari Waweru-Siika, Fernando Zampieri.

Supplementary table 1. Covariates which had missing data which were singly imputed using random forest

Variable	Survival model<br>n imputed (of N = 3070)	Multi state model<br>n imputed (of N=3778)
**Sex**	9	10
**Age**	--	6
**Comorbidities**	123	131
**COVID vaccination**	1	505
**Oxygen saturation at admission**	9	-
**Oxygen device at admission**	18	10

Supplementary table 2. Baseline characteristics and oxygen support modality by region

	N	Overall, N = 3,070[1]	Africa, N = 861	Americas, N = 284	Eastern Mediterranean, N = 543	Europe, N = 327	South-East Asia, N = 611	Western Pacific, N = 444	p-value[2]
**Demographics**									
Age (years), median (IQR)	3,066	63 (47, 73)	50 (37, 65)	70 (57, 81)	66 (52, 75)	70 (62, 79)	62 (50, 71)	65 (52, 78)	<0·001
Sex, n (%)	3,061								0·052
Female		1,471 (48%)	391 (45%)	129 (46%)	264 (49%)	181 (56%)	294 (48%)	212 (48%)	
Male		1,590 (52%)	470 (55%)	154 (54%)	273 (51%)	144 (44%)	317 (52%)	232 (52%)	
Body mass index (BMI), n (%)	2,886								<0·001
Underweight (<18·5)		205 (7·1%)	39 (5·0%)	30 (12%)	13 (2·5%)	7 (2·2%)	62 (10%)	54 (13%)	
Normal weight (18·5 - 24·9)		1,248 (43%)	352 (45%)	105 (41%)	138 (27%)	98 (30%)	353 (58%)	202 (49%)	
Pre-obese (25 - 29·9)		888 (31%)	282 (36%)	68 (27%)	187 (36%)	109 (34%)	145 (24%)	97 (24%)	
Obese (>= 30)		545 (19%)	101 (13%)	51 (20%)	176 (34%)	108 (34%)	51 (8·3%)	58 (14%)	
Pregnancy status, n (%)	1,467	15 (1·0%)	9 (2·3%)	0 (0%)	3 (1·1%)	0 (0%)	3 (1·0%)	0 (0%)	0·053
Vaccinated, n (%)	3,069								<0·001
Yes		1,683 (55%)	295 (34%)	144 (51%)	289 (53%)	117 (36%)	513 (84%)	325 (73%)	
No		966 (31%)	384 (45%)	19 (6·7%)	196 (36%)	188 (57%)	86 (14%)	93 (21%)	
Unknown		420 (14%)	182 (21%)	120 (42%)	58 (11%)	22 (6·7%)	12 (2·0%)	26 (5·9%)	
**Underlying conditions**									
Number of underlying conditions, n (%)	2,947								<0·001
No underlying condition		330 (11%)	92 (12%)	24 (8·8%)	50 (9·5%)	19 (5·8%)	110 (18%)	35 (8·2%)	
One comorbidity		803 (27%)	266 (34%)	82 (30%)	121 (23%)	42 (13%)	187 (31%)	105 (25%)	
Two or more comorbidities		1,814 (62%)	428 (54%)	167 (61%)	356 (68%)	266 (81%)	310 (51%)	287 (67%)	
Chronic cardiac disease, n (%)	2,975	716 (24%)	110 (13%)	50 (19%)	160 (30%)	170 (52%)	91 (15%)	135 (32%)	<0·001
Hypertension, n (%)	3,006	1,539 (51%)	335 (40%)	137 (51%)	305 (56%)	254 (79%)	253 (42%)	255 (60%)	<0·001
Chronic obstructive pulmonary disease, n (%)	2,983	511 (17%)	73 (8·7%)	39 (15%)	80 (15%)	34 (11%)	230 (38%)	55 (13%)	<0·001
Asthma, n (%)	2,984	255 (8·5%)	105 (13%)	20 (7·6%)	35 (6·5%)	17 (5·3%)	35 (5·8%)	43 (10%)	<0·001
Chronic liver disease, n (%)	2,992	105 (3·5%)	34 (4·1%)	3 (1·1%)	22 (4·1%)	8 (2·5%)	13 (2·1%)	25 (5·8%)	0·005
Dementia, n (%)	2,978	112 (3·8%)	31 (3·8%)	23 (8·6%)	19 (3·6%)	21 (6·5%)	7 (1·1%)	11 (2·5%)	<0·001

	Chronic neurological disease, n (%)	3,002	230 (7·7%)	36 (4·3%)	33 (13%)	57 (11%)	43 (13%)	19 (3·1%)	42 (9·7%)	<0·001
	HIV, n (%)	2,827	198 (7·0%)	147 (20%)	29 (11%)	0 (0%)	5 (1·6%)	2 (0·3%)	15 (3·5%)	<0·001
	Diabetes, n (%)	2,986	780 (26%)	148 (18%)	74 (28%)	219 (41%)	104 (32%)	158 (26%)	77 (18%)	<0·001
	Current smoking, n (%)	2,89	408 (14%)	92 (12%)	23 (9·1%)	83 (17%)	48 (15%)	114 (19%)	48 (11%)	<0·001
	Tuberculosis, n (%)	2,945	189 (6·4%)	82 (10%)	19 (7·4%)	10 (1·9%)	3 (0·9%)	43 (7·2%)	32 (7·6%)	<0·001
	Cancer, n (%)	2,989	158 (5·3%)	39 (4·7%)	13 (4·9%)	38 (7·2%)	35 (11%)	12 (2·0%)	21 (4·8%)	<0·001
**Vital signs**										
	Heart rate beats/min, Median (IQR)	3,066	90 (79, 108)	98 (84, 113)	85 (76, 101)	89 (79, 103)	84 (76, 97)	100 (86, 110)	86 (75, 96)	<0·001
	Respiratory rate breaths/min, Median (IQR)	2,877	24 (20, 29)	28 (23, 34)	20 (18, 22)	22 (20, 26)	20 (18, 23)	25 (22, 30)	23 (21, 26)	<0·001
	Systolic blood pressure mmHg, Median (IQR)	3,049	128 (110, 140)	125 (108, 146)	127 (112, 140)	126 (110, 140)	130 (115, 140)	130 (110, 143)	123 (114, 138)	0·056
	FiO$_2$, Median (IQR)	2,451	40 (28, 44)	40 (32, 40)	32 (28, 40)	40 (28, 60)	40 (36, 40)	36 (28, 50)	40 (32, 50)	<0·001
	Oxygen saturation, median (IQR)	3,061	94·0 (90·0, 97·0)	91·0 (86·0, 96·0)	95·0 (92·0, 97·0)	94·0 (91·0, 96·0)	95·0 (92·0, 97·0)	95·0 (91·0, 97·0)	96·0 (90·0, 98·0)	<0·001
	SpO$_2$ / FiO$_2$, median (IQR)	2,443	243 (196, 315)	235 (213, 275)	291 (230, 346)	238 (162, 321)	238 (224, 265)	266 (187, 343)	240 (194, 297)	<0·001
	Decreased level of consciousness, n (%)		1000 (33%)	380 (44%)	95 (33%)	104 (19%)	229 (70%)	116 (19%)	76 (17%)	<0·001
**Oxygen support modality**										
	Oxygen support modality, n (%)	3,052								
	None (room air)		316 (10%) [3]	103 (12%)	32 (11%)	63 (12%)	28 (8·6%)	74 (12%)	16 (3·6%)	
	Nasal cannula		1,394 (46%)	550 (64%)	159 (56%)	155 (29%)	95 (29%)	187 (31%)	248 (56%)	
	Simple face mask		687 (23%)	69 (8·1%)	5 (1·8%)	147 (27%)	165 (50%)	254 (42%)	47 (11%)	
	Venturi mask		67 (2·2%)	6 (0·7%)	10 (3·5%)	27 (5·0%)	2 (0·6%)	7 (1·2%)	15 (3·4%)	
	Non-rebreather mask		265 (8·7%)	97 (11%)	25 (8·8%)	41 (7·6%)	4 (1·2%)	43 (7·1%)	55 (12%)	
	HFNO		49 (1·6%)	1 (0·1%)	7 (2·5%)	8 (1·5%)	2 (0·6%)	7 (1·2%)	24 (5·4%)	
	CPAP		38 (1·2%)	4 (0·5%)	2 (0·7%)	13 (2·4%)	11 (3·4%)	4 (0·7%)	4 (0·9%)	
	NIV		65 (2·1%)	0 (0%)	4 (1·4%)	43 (7·9%)	5 (1·5%)	12 (2·0%)	1 (0·2%)	
	Invasive mechanical ventilation		171 (5·6%)	23 (2·7%)	40 (14%)	45 (8·3%)	15 (4·6%)	14 (2·3%)	34 (7·7%)	

[1] Median (IQR); n (%)

[2] Statistical testing was by Kruskal-Wallis rank sum test (for continuous variables), and Fisher's exact test (for proportions)

[3] This group (not receiving oxygen) comprised: 91 with SpO$_2$ <92 but were not receiving supplemental oxygen; 60 with SpO$_2$ 92-94% with danger sign but were not receiving supplemental oxygen; 165 who after screening did not require oxygen therapy at the point of initial detailed data capture.

Supplementary table 3. Respiratory support at time of enrolment

	Nasal cannula	Simple face mask	Venturi mask	Non-rebreather mask	HFNO	CPAP	BiPAP	Invasive mechanical ventilation
**Characteristic**	**N = 1,376**[1]	**N = 687**[1]	**N = 67**[1]	**N = 265**[1]	**N = 49**[1]	**N = 38**[1]	**N = 65**[1]	**N = 171**[1]
**Flow**								
1-5 L/m	1,181 (86%)	327 (48%)	8 (12%)	14 (5%)	0 (0%)	1 (5%)	14 (26%)	6 (15%)
6-10 L/m	167 (12%)	306 (45%)	43 (64%)	100 (38%)	0 (0%)	4 (21%)	18 (33%)	2 (5%)
11-15 L/m	6 (0%)	42 (6%)	16 (24%)	139 (52%)	1 (2%)	1 (5%)	17 (31%)	5 (13%)
>15 L/m	19 (1%)	11 (2%)	0 (0%)	12 (5%)	41 (98%)	13 (68%)	5 (9%)	27 (68%)
**Fraction of inspired oxygen (%)**	36 (32, 40)	50 (40, 55)	50 (50, 60)	95 (80, 95)	60 (50, 90)	60 (50, 70)	52 (40, 80)	60 (40, 100)
**Peak airway pressure (cm)**								23 (18, 30)
**Positive end-expiratory pressure (cm)**								6 (5, 8)

[1]n (%); Median (IQR)

Supplementary table 4. Length of hospital stay and highest level of oxygen support by region

	All N = 3,070	AFR N = 861	AMR N = 284	EMR N = 543	EUR N = 327	SEAR N = 611	WPR N = 444
**None (room air), n (%)**	232 (7·6%)	86 (10%)	24 (8·5%)	38 (7·0%)	24 (7·3%)	53 (8·7%)	7 (1·6%)
**Nasal cannula or Venturi or simple mask, n (%)**	1,964 (64%)	607 (71%)	148 (52%)	288 (53%)	225 (69%)	440 (72%)	256 (58%)
**Non-rebreather mask, n (%)**	287 (9·4%)	132 (15%)	16 (5·6%)	37 (6·8%)	5 (1·5%)	47 (7·7%)	50 (11%)
**HFNO, n (%)**	79 (2·6%)	2 (0·2%)	9 (3·2%)	12 (2·2%)	9 (2·8%)	10 (1·6%)	37 (8·3%)
**CPAP or NIV, n (%)**	166 (5·4%)	7 (0·8%)	12 (4·2%)	72 (13%)	17 (5·2%)	37 (6·1%)	21 (4·7%)
**IMV, n (%)**	340 (11%)	26 (3·0%)	75 (26%)	95 (18%)	47 (14%)	24 (3·9%)	73 (16%)

** p <0·001*

AFR Africa; AMR Americas; EMR Eastern Mediterranean; EUR Europe; SEAR South-East Asia; WPR Western Pacific

CPAP = continuous positive airway pressure; HFNO = high flow nasal oxygen; IMV = invasive mechanical ventilation; NIV = non-invasive ventilation

Supplementary table 5. Basis of calculations of FiO_2

	Formula	value restriction	FiO2 range
**Value available from interface (e.g. Venturi mask, NIV, IMV)**	value		0·21 – 1·0
**Room air**	0·21		0·21
**Nasal cannula**	0·20 + 0·04 * flow_rate	ceiling = 0·44	0·24 – 0·44
**Face mask**	5L/min = 0·4<br>6L/min = 0·5<br>7L/min = 0·55<br>8L/min = 0·6	floor = 0·4<br>ceiling = 0·6	0·4 – 0·6
**Non-rebreather mask**	0·1 * flow_rate	floor = 0·6<br>ceiling = 0·95	0·6 – 0·95

Supplementary table 6. Summary of ethical approvals for the study

Country	Ethics board (REC/IRB)	REC reference	Allowed waiver of written consent	Allowed oral consent in place of written	Required minor assent in addition to parental consent
**Bangladesh**	Chittagong Medical College ERC	CMC/PG/ 2021/ 175	No	No	No
**Brazil**	Instituto Nacional de Infectologia Evandro Chagas - INI/Fiocruz	53800821. 3.0000.5262	No	No	No
**Colombia**	Comité de Ética en Investigación de la Fundación Universitaria Sanitas	CEIFUS 2179-21	Yes	N/A	N/A
**Democratic Republic of Congo**	University of Kinshasa School of Public Health Ethics Committee	ESP/CE/ 184/ 2021	No	No	Yes
**Egypt**	Suez Canal University Faculty of Medicine REC	4776	No	No	No
**El Salvador**	Hospital El Salvador Bioethics Committee and Research Ethics	-	Yes	N/A	N/A
**India**	Father Muller Institutional EC	FMIEC/CCM/ 1030/ 2021	Yes	N/A	N/A
**India**	JSS Medical College Institutional EC	17112021/ 43NCT/ 2021-22	Yes	N/A	N/A
**Iran**	Tabriz University of Medical Sciences REC	IR.TBZMED. REC.1400.739	Yes	Yes	N/A
**Jordan**	Jordan University of Science and Technology REC	11/144/2021	Yes	N/A	N/A
**Kazakhstan**	Bioethics Committee Karaganda Medical University	2/2/5	Yes	N/A	N/A
**Lebanon**	Bellevue Medical Center IRB	-	No	No	Yes
**Lebanon**	Hotel-Dieu de France Ethics Committee	CEHDF 1925	Yes	N/A	N/A
**Malawi**	Malawi College of Medicine REC	P.11/ 21/ 3464	No	No	Yes
**Mongolia**	Mongolian National University of Medical Sciences IRB	44265	No	No	No
**Nepal**	Nepal Health Research Council	42/ 2022P	No	Yes	No
**Nepal**	Nepal Health Research Council	676/ 2021P	No	No	No
**Nigeria**	Ogun State REC	HPRS/ 381/ 418	Yes	N/A	N/A
**Nigeria**	University of Calabar TH Health Research Ethics Committee	UCTH/HREC/ 33/ Vol.111/ 033	Yes	N/A	N/A
**Pakistan**	Aga Khan University Ethics Review Committee	2021-6880-19620	Yes	N/A	N/A
**Pakistan**	Health Research Institute National Bioethics Committee, Ziauddin University ERC	No.4-87/ NBC/ COVID-95/ 21/ 671, 4321021AKPUL	Yes	N/A	N/A
**Peru**	Universidad Peruana Cayetano	132 - 01 - 22	Yes	N/A	N/A
**Philippines**	Lung Center of the Philippines IRB	LCP-CS-014-2022	Yes	N/A	N/A
**Philippines**	Davao Center for Health Development Joint REC	JREC-2212	No	No	No
**Republic of Moldova**	National Committee for Ethical Expertise of Clinical Trial	NCEECT/ 1248	No	No	No
**Serbia**	Klinicko Bolnicku Centar Bezanijska Kosa	1268/1	Yes	N/A	N/A
**South Africa**	University of Kwazulu-Natal	BREC/ 00004283/ 2022	No	No	Yes
**South Africa**	University of Pretoria, Faculty of Health Sciences REC	15/ 2022	No	No	Yes
**Uganda**	Uganda National Council for Science and Technology	HS2399ES	No	No	Yes
**Viet Nam**	National Hospital for Tropical Diseases REC	1141/ QD-BVBND	Yes	N/A	N/A

Supplementary table 7. Unadjusted hazard ratios (HR) for the mode of oxygen delivery and region

A: Unadjusted hazard ratios for region (adjusted HR given in the main manuscript):

Characteristic	log(HR)	95% CI	p-value
**WHO region**			
**Europe**	—	—	
**Africa**	0.91	0.62, 1.2	<0.001
**Americas**	0.21	-0.15, 0.57	0.3
**Eastern Mediterranean**	0.51	0.19, 0.84	0.002
**South-East Asia**	-0.06	-0.45, 0.32	0.7
**Western Pacific**	-0.02	-0.37, 0.33	>0.9

B: Unadjusted hazard ratios for oxygen device at day 1 (adjusted HR given in the main manuscript):

Characteristic	log(HR)	95% CI	p-value
**O2 device at admission**			
**None (room air)**	—	—	
**Nasal cannula**	0.08	-0.24, 0.41	0.6
**Simple face mask**	0.04	-0.32, 0.39	0.8
**Venturi mask**	-0.11	-0.75, 0.53	0.7
**Non-rebreather mask**	0.78	0.42, 1.1	<0.001
**HFNO**	0.15	-0.46, 0.75	0.6
**CPAP**	0.62	0.03, 1.2	0.039
**Non-invasive ventilation**	0.62	0.08, 1.2	0.024
**Invasive ventilation**	0.93	0.57, 1.3	<0.001

Abbreviations: CI = Confidence Interval, HR = Hazard Ratio

Supplementary figure 1. Distribution of oxygen requirement per patient during first seven days of admission

Daily oxygen usage is based on delivery modality and flow rate (see Supplementary table 5). Error bars represent IQR, with whiskers extending to 1·5 x IQR from the box, and outliers displayed as closed circles.

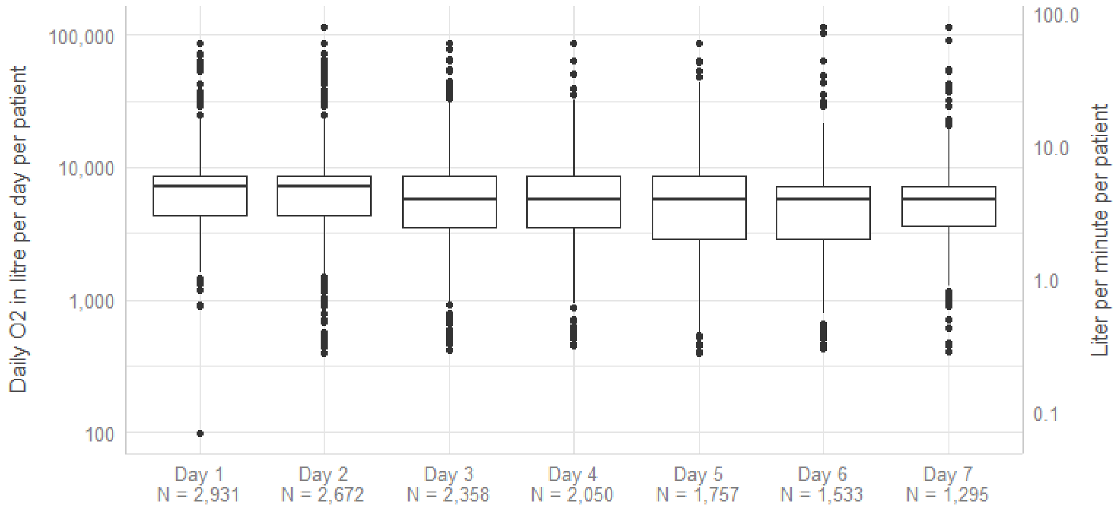

Supplementary figure 2. Distribution of daily oxygen per day of follow up stratified by oxygen delivery mode at admission

Daily oxygen usage is based on delivery modality and flow rate (see Supplementary table 5). Error bars represent IQR, with whiskers extending to 1·5 x IQR from the box, and outliers displayed as closed circles.

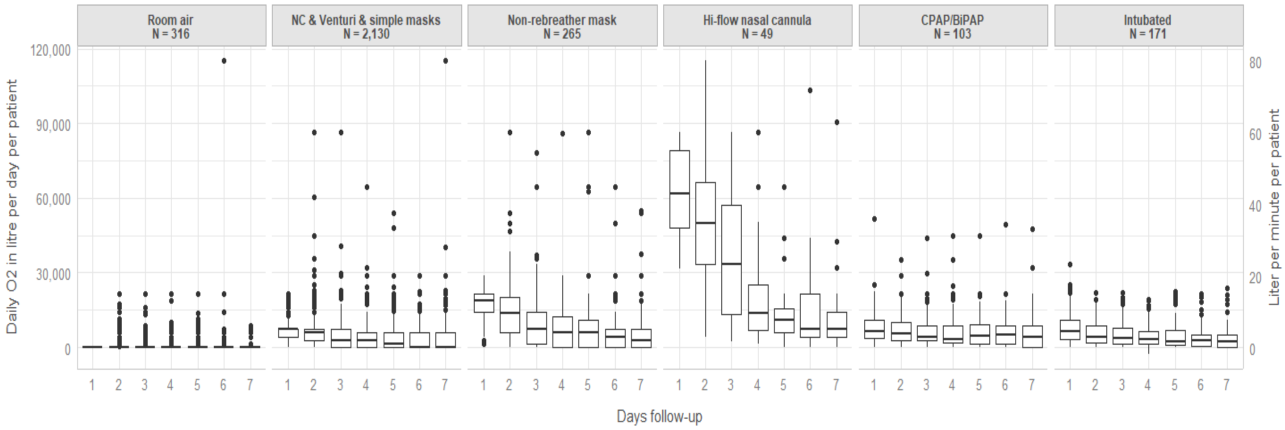

CPAP = continuous positive airway pressure; HFNO = high flow nasal oxygen; IMV = invasive mechanical ventilation; NIV = non-invasive ventilation

Supplementary figure 3. Daily oxygen consumption by type of respiratory support

Median daily oxygen consumption for patients based on delivery modality and flow rate (see Supplementary table 5). Error bars represent IQR, with whiskers extending to 1·5 x IQR from the box, and outliers displayed as closed circles.

Patients on high-flow nasal oxygen and non-rebreather masks had higher median daily oxygen requirements, with values of 21,144 L and 17,280 L respectively.

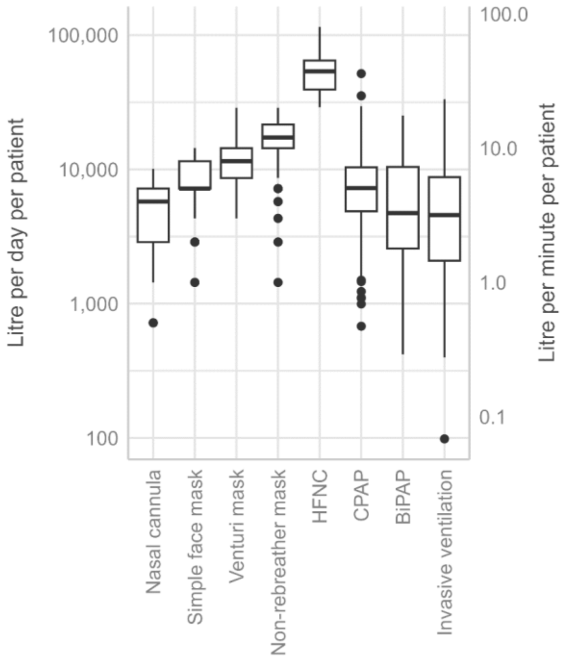

CPAP = continuous positive airway pressure; HFNO = high flow nasal oxygen; IMV = invasive mechanical ventilation; NIV = non-invasive ventilation

Supplementary figure 4. Trajectory of oxygen support levels and associated outcomes

Likelihood of patients transitioning through increasing states of respiratory support within the multistate Markov model are described by adjusted hazard ratios (aHR) with 95% confidence intervals.

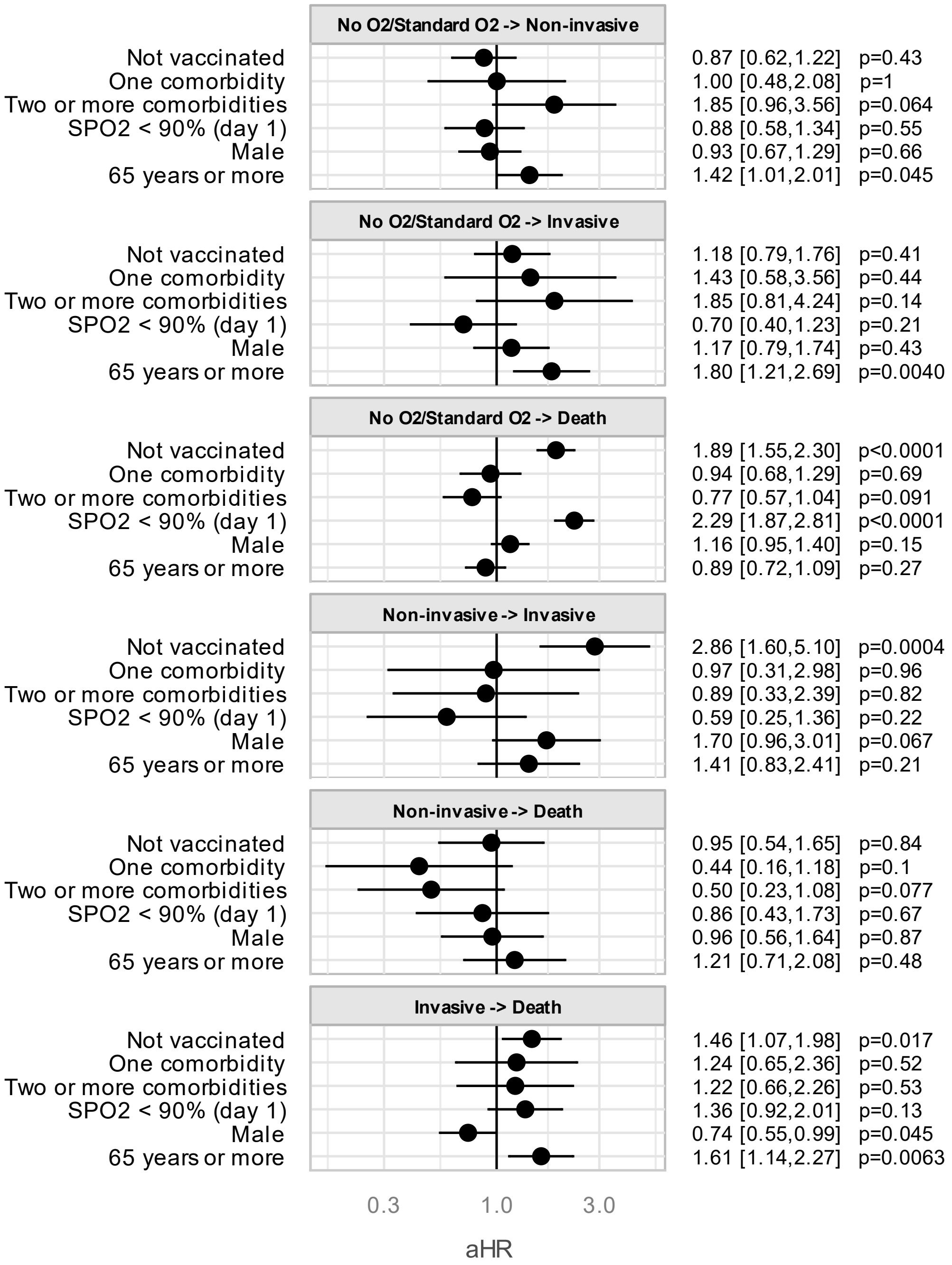

Supplementary figure 5. Electricity source by region

Sources of electricity by region expressed as (A) bar chart and (B) radar plot. Each site may have multiple sources of oxygen.

A

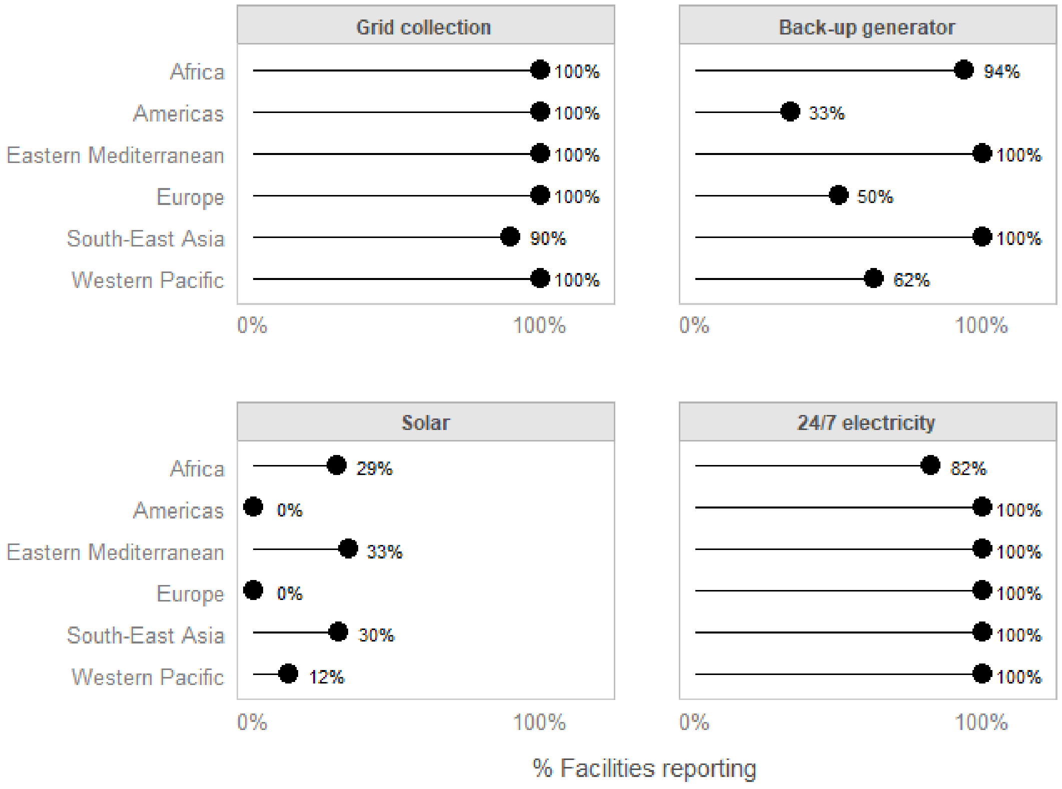

B

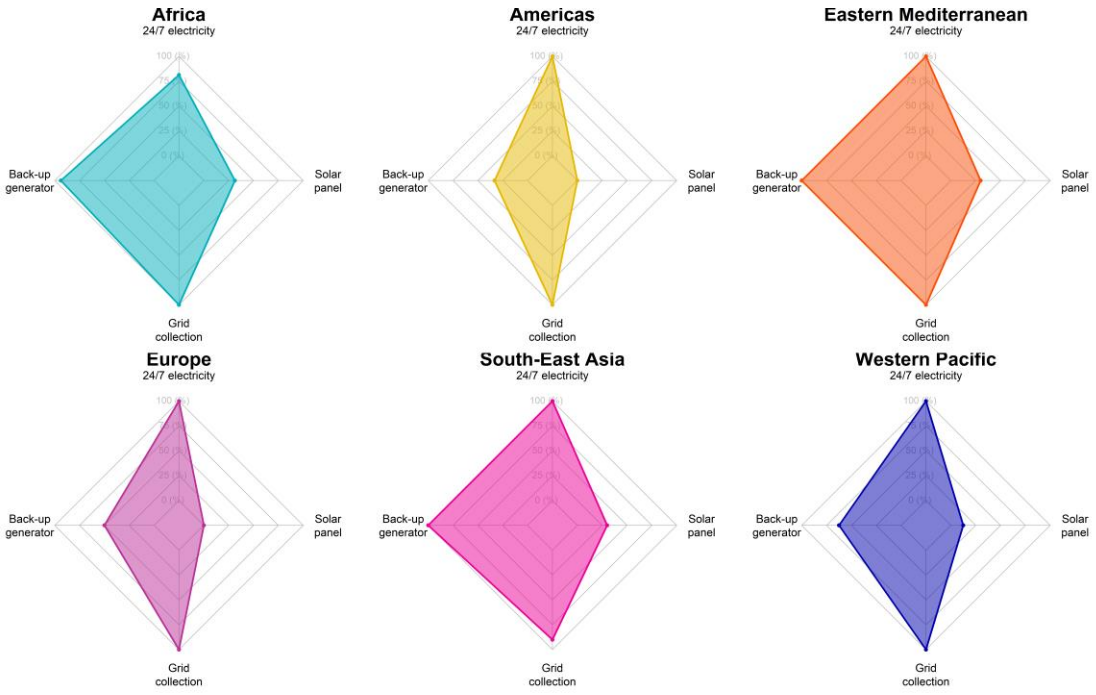

Supplementary figure 6. Oxygen source by region (radar plot)

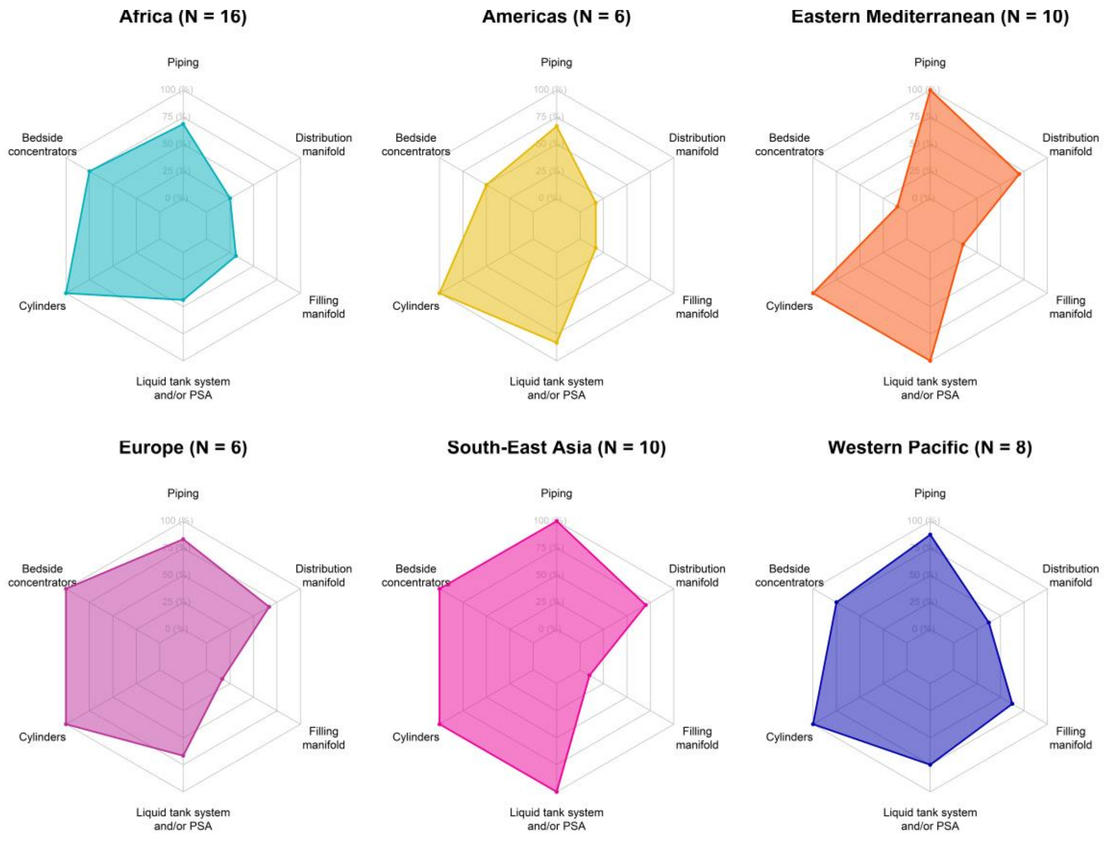

Supplementary figure 7. Trajectory of oxygen support modality and associated outcomes

Within the multistate model, proportions next to linking arrows depict the proportion and number of patients transitioning between states.

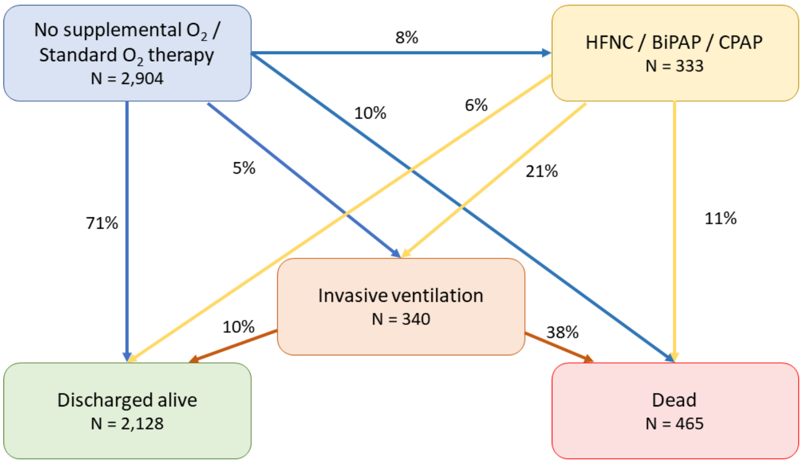

Supplement: Supplementary appendix [file mmc1.pdf]
